# Signalling pathways in autism spectrum disorder: mechanisms and therapeutic implications

**DOI:** 10.1038/s41392-022-01081-0

**Published:** 2022-07-11

**Authors:** Chen-Chen Jiang, Li-Shan Lin, Sen Long, Xiao-Yan Ke, Kohji Fukunaga, Ying-Mei Lu, Feng Han

**Affiliations:** 1 International Joint Laboratory for Drug Target of Critical Illnesses; Key Laboratory of Cardiovascular & Cerebrovascular Medicine, School of Pharmacy, Nanjing Medical University, Nanjing, 211166 China; 2 Department of Physiology, School of Basic Medical Sciences, Nanjing Medical University, Nanjing, 211166 China; 3 Department of Pharmacy, Hangzhou Seventh People’s Hospital, Mental Health Center Zhejiang University School of Medicine, Hangzhou, 310013 China; 4 Child Mental Health Research Center, Nanjing Brain Hospital, Nanjing Medical University, Nanjing, 210029 China; 5 Department of CNS Drug Innovation, Graduate School of Pharmaceutical Sciences, Tohoku University, Sendai, 980-8578 Japan; 6 Institute of Brain Science, The Affiliated Brain Hospital of Nanjing Medical University, Nanjing, 210029 China; 7 Gusu School, Nanjing Medical University, Suzhou Municipal Hospital, The Affiliated Suzhou Hospital of Nanjing Medical University, Suzhou, 215002 China

**Keywords:** Neuroscience, Molecular biology

## Abstract

Autism spectrum disorder (ASD) is a prevalent and complex neurodevelopmental disorder which has strong genetic basis. Despite the rapidly rising incidence of autism, little is known about its aetiology, risk factors, and disease progression. There are currently neither validated biomarkers for diagnostic screening nor specific medication for autism. Over the last two decades, there have been remarkable advances in genetics, with hundreds of genes identified and validated as being associated with a high risk for autism. The convergence of neuroscience methods is becoming more widely recognized for its significance in elucidating the pathological mechanisms of autism. Efforts have been devoted to exploring the behavioural functions, key pathological mechanisms and potential treatments of autism. Here, as we highlight in this review, emerging evidence shows that signal transduction molecular events are involved in pathological processes such as transcription, translation, synaptic transmission, epigenetics and immunoinflammatory responses. This involvement has important implications for the discovery of precise molecular targets for autism. Moreover, we review recent insights into the mechanisms and clinical implications of signal transduction in autism from molecular, cellular, neural circuit, and neurobehavioural aspects. Finally, the challenges and future perspectives are discussed with regard to novel strategies predicated on the biological features of autism.

## Introduction

**Fig. 1:**
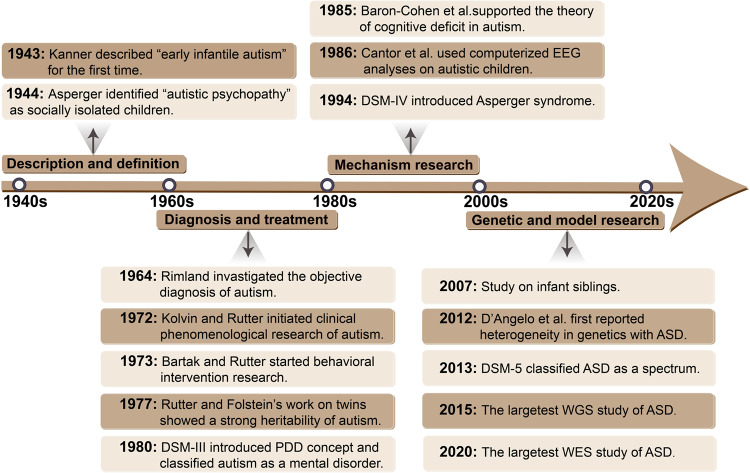
The milestone events associated with autism. Original description of autism was in 1940s, subsequently leading to a series of studies on the definition, diagnosis and treatment of autism in 1960s and 1970s. From the first twin study in 1977, people began to realize that autism as a common highly heritable neurodevelopmental disorder. Up to now, advances in WGS and WES have revealed patterns of inheritance and the types of genetic variation that result in ASD and studies in models have identified a mountain of evidence for molecular mechanisms for ASD. PDD pervasive developmental disorder, EEG electroencephalography, WGS whole gene sequencing, WES whole-exome sequencing

Autism spectrum disorder (ASD), a group of early developmental disorders, is characterized by deficits in social communication and repetitive stereotyped behaviours. Over the past 80 years, risk factors, diagnostic criteria, clinical treatment options, and societal implications of ASD have attracted the concerns of neuroscientists and clinicians (Fig. 1).

In 1943, Leo Kanner of Johns Hopkins University published “Autistic disturbances of affect contact” in the special issue of the journal *The Nervous Child*, which systemically examined 11 cases of autism and named it “early infantile autism”.^1^ Kanner used the term ‘infantile autism’ to describe the children with symptoms of social isolation and linguistic disorders. However, some aspects of Kanner’s views also called the origin of early confusion in the field, such as the vague definition between schizophrenia and autism.^2^ In 1944, Hans Asperger identified a group children have severe social abnormalities and motor disorders but with very high intellectual functioning.^3^ This led to the diagnosis of high-functioning autism, that has been incorporated into the *Diagnostic and Statistical Manual of Mental Disorders, 4th edition* (DSM-IV) and the 10th edition of the World Health Organization’s *International Statistical Classification of Diseases and Related Health Problems* (ICD-10) and named “Asperger’s Syndrome”.^4–6^

In the 1960s and 1970s, early pioneering works on the diagnosis and treatment of autism were initiated. In 1964, Bernard Rimland first began to investigate new approaches to the objective diagnosis of autism.^7^ In 1972, based on studies of clinical phenomenology, Rutter made clear that autism has significant differences from schizophrenia in terms of onset, clinical symptoms, and family history.^8^ Rutter’s research also suggested that it would be more plausible to attribute autistic behaviours to developmental disorders from birth to early childhood. By the late 1970s, a consensus emerged about the importance of studying autism independently of schizophrenia, which promoted the updating of diagnostic criteria.^9,10^ In 1978, Rutter proposed new diagnostic criteria for autism emphasizing social skill dysfunction, language and communication impairment, and repetitive behaviours as three aspects of the basic criteria, abandoning the “special skills and attractive appearance” of Kanner’s criteria.^9^ The diagnostic approach provided by Rutter directly influenced the revision of DSM-III. In 1980, DSM-III first regarded “infantile autism” as a pervasive developmental disorder (PDD) and focused on early development. Over the same period, studies on intervention and treatment also greatly improved. In 1973, Bartak and Rutter recommended the importance of a structured, behavioural improvement-focused treatment plan.^11^ Subsequently, an increasing number of behavioural intervention studies have supported the notion that behavioural psychology and special education can be applied to inform autism therapy.

In the 1980s, autism research entered a new era, especially in terms of mechanisms. Autism gradually began to be viewed as a somatic developmental disorder unrelated to parenting styles. Researchers began exploring the aetiology of autism from a biological perspective and completely distinguished autism from schizophrenia on account of clinical symptom recognition and clinical diagnosis. In 1977, Folstein and Rutter’s first study on twins revealed the high heritability of autism.^12^ Subsequently, with the in-depth understanding of autism, people gradually realized that autism is a developmental disorder under the influence of certain genetic factors.^13,14^ On this foundation, substantial research into the genesis of autism has been conducted, including molecular genetics, neuroimmunity, functional imaging, neuroanatomy, and neurochemistry research.

ASD is considered to be the result of complex interactions among genetic, environmental, and immunological factors.^15–17^ There have been incredible improvements in the investigation of genetic correlations with autism over the past two decades, ranging from monoclonal gene studies^18^ to contemporary large-scale studies using whole-genome sequencing (WGS).^19^ A number of highly reliable and repetitive risk genes have been discovered.^20,21^ Based on studies of genetically modified mice, considerable progress has been made in illustrating the functions of genes such as *Mecp2* (Rett syndrome), *Tsc1/2* (tuberous sclerosis), *Fmrp* (fragile X syndrome), *Pten* and *Shank3* (Phelan–McDermid syndrome) in several monogenetic diseases. These advances in disease mechanism research provide the basis for the design of drugs such as rapamycin (mTOR) inhibitors (tuberous sclerosis^22^ and fragile X syndrome,^23,24^) metabolic glutamate receptor (mGluR) antagonists (fragile X syndrome^25^ and 16p11.2 deletion^26^), and insulin growth factor (IGF-1) (Rett syndrome^27^ and Phelan–McDermid syndrome^28,29^).

In addition to the downregulation of synapse-related genes, microglia and immune-related genes were increased in the brains of autistic patients.^30–32^ The correlations among astrocytes, microglial activation, neuroinflammation caused by gut microbiota and immune dysregulation in ASD patients are also involved in the pathological mechanism.^17,33–36^ In particular, infection during pregnancy has been established to induce maternal immune activation that affects the offspring nervous system.^37,38^

Another pathological mechanism of ASD that has garnered much attention is the functional impairment of brain regions and neural circuits. Autopsies of patients with ASD have revealed significant structural changes in their brains, including altered grey/white matter ratios, increased neuronal numbers, decreased neuronal body volume, increased numbers of glia, and changes in dendritic spines and cerebral blood vessels.^39^ Additionally, there is established evidence of alterations in glutamate circuits and GABAergic circuits in ASD patients, as manifested by increased numbers of excitatory synapses and spine densities, significantly reduced levels of glutamic acid decarboxylase, and GABAA and GABAB receptor alterations in the postmortem brains of patients with autism.^40,41^

In this review, we integrate recent advances from genetic, neuropathological, and neurobiochemical studies on ASD to further elucidate the pathogenetic mechanism at the molecular, cellular, and neural circuit levels.

## Clinical overview and genetic features

### Definition and diagnosis of ASD

Since autism was discovered 80 years ago, its clinical definition and diagnostic criteria have undergone several iterations. In 1980, the DSM-III classified “infantile autism” as one of the generic “PDDs”.^42^ In 1994, five PDDs were included in the DSM-IV: autism disorder, Asperger’s syndrome, PDD-not otherwise specified (PDD-NOS), Rett syndrome and childhood disintegrative disorder.^5^ Given the large variability in symptom severity across disease groups, it is difficult to effectively distinguish diseases. To remove this uncertainty, the DSM-5 classifies autism, Asperger’s syndrome, and PDD-NOS as ASD.^43^ With this revision, the diagnostic criteria have changed as well. ASD is characterized by two main symptoms: deficits in social interaction/communication, as well as repetitive stereotyped behaviours that first occur in early developmental stages and cause clinically substantial impairment.^44^ Aside from the core features above, individuals with ASD are frequently associated with co-occurring symptoms, including dyskinesia (hypotonia, bradykinesia), speech delay, sleep disorder, gastrointestinal problems, anxiety and epilepsy, which are the most common symptoms in preschool children, while in adolescents and adults, the proportion of depressive symptoms is higher.^45–47^ These comorbidities also pose challenges to disease modelling of ASD, as they may complicate the evaluation of ASD core behaviours in animal models.

The diagnosis of autism is based on thorough consideration of medical history, physical and neurological examination, psychiatric examination, and auxiliary examinations.^48^ A comprehensive review of the family history of ASD or other neurological disorders should also be included. Autism diagnoses from preschool to mid-childhood are highly stable.^49^ Due to the complexity, severity, and overlap of ASD features, the correct diagnosis of ASD with instruments and scales is essential for improving the clinical management of patients. Several scales have been suggested that can be helpful for identifying ASD.^50^

### Epidemiology of ASD

Over the past two decades, the prevalence of ASD reported worldwide has been steadily increasing. In 2000, according to the Autism and Developmental Disabilities Monitoring (ADDM), the incidence of ASD was estimated to be 1 in 150 children. In 2006, the incidence was 1 in 110 children, and by 2008, the incidence had increased to 1 in 88 children.^50^ According to recent estimates, more than 70 million people worldwide have suffered from autism, and the overall estimated prevalence is between 1.5% and 2%.^51,52^ Modifications in diagnostic criteria and increased awareness of autism in people might be responsible for the surge in autism. Estimates of autism prevalence in different populations and settings vary by definition, sampling, and assessment of independent population cases among studies.

Notably, there is a prominent sex difference in the prevalence of ASD, with prevalences of 2.8% in males and 0.65% in females and a male-to-female ratio of 4.3:1.^51,52^ This suggests that unknown biological factors may play a role.^53–56^ Moreover, a recent study showed an increased female-to-male odds ratio for ASD comorbidities and showed that comorbidity occurrence was associated with the age at first autism diagnosis.^57^ There may be differences in gene expression induced by gonadal hormones or sex chromosomes in mammals.^58^ In the brain, more genes are expressed from the X chromosome than from the Y chromosome. The mutations in the X chromosome are generally associated with intellectual disability syndrome, which is more prevalent in males than in females.^59,60^ The earliest studies on the rare variant of ASD have also tended to focus on the contributions of chromosomal abnormalities in girls. A rare *LGD* mutation has been found in the *NLGN4* and *NLGN3* genes, both of which are located on the X chromosome.^61^ As an X-linked neurodevelopment disorder, Rett syndrome almost exclusively influences females. One possibility is that mutations in Rett syndrome occur almost exclusively on the paternally derived X chromosome and are lethal in male embryos.^62^ In general, the contribution of gender aetiology to autism remains largely unexplained. Human studies have only identified minor sex variations in cerebral cortical gene expression.^63–66^ Resolving sex differences is a significant aspect in the process of ASD and shows great potential for the development of widely applicable therapies. Many psychiatric disorders, including ASD, will probably be better understood if key sex differences in cellular and molecular events during brain differentiation can be identified.

### Genetic architecture of ASD

**Fig. 2:**
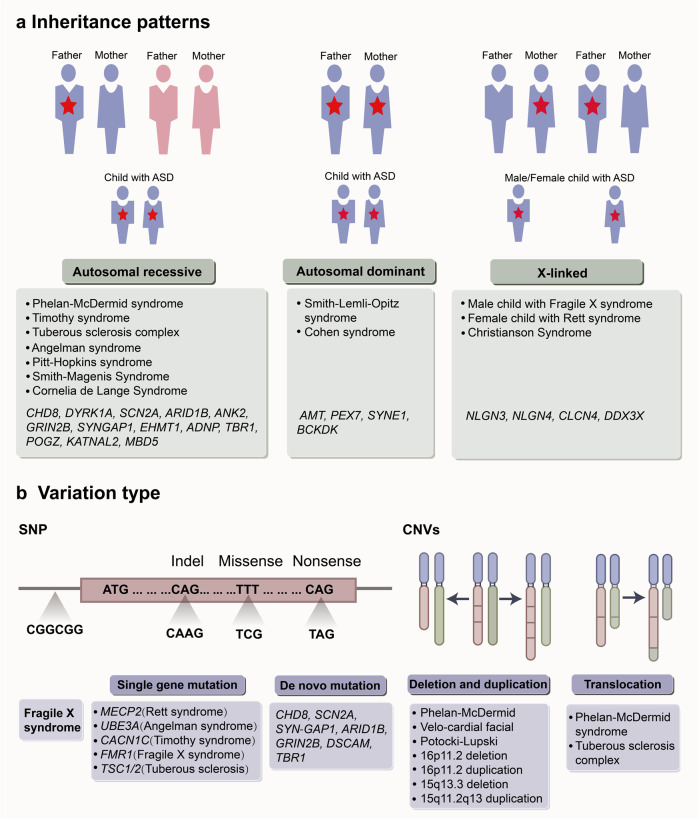
Genetic architecture of autism spectrum disorder (ASD). **a** The inheritance patterns of high-risk gene and syndromes associated with ASD. Major gene model includes autosomal recessive, autosomal dominant and X-linked inheritance patterns. The red stars indicate a causal allele. **b** The shown types of genetic variation including SNP and CNVs. Genes and syndrome that have been associated with ASD are also indicated. SNP single-nucleotide polymorphisms, CNV copy number variation. (Adapted with permission from reference^15^)

Twin and family studies have consistently suggested that autism have a strong heritability.^14,67,68^ Recent advances in genetic technology, microarrays, WGS, and whole-exome sequencing (WES) have revealed patterns of genetic variation that result in ASD.^19,69,70^ Here, we highlight the contributions of inheritance patterns, variation types and epidemic rates to ASD (Fig. 2). Heritability measurements have been derived from investigations on identical twins, fraternal twins and sibling concordance, including a survey of more than 2 million Swedish households in 2014,^71^ which is the largest human-based ASD study to date, eventually estimating the heritability of ASD as ranging from 52% to 90%.^68,72,73^ Moreover, the epidemiological and molecular data suggest that the genetic contribution of ASD results from the combination of rare deleterious variants and a large number of low-risk alleles.^74^ Therefore, different phenotypes can arise because prevalent low-risk alleles buffer the effects of detrimental variantion.^74–76^

The genetic structure of ASD is extremely complex. Approximately 600–1200 genes and genomes have been identified that associated with autism.^77^ At least 5% of ASD cases are caused by single-nucleotide polymorphisms (SNPs) in genes such as *NLGN3, NLGN4, NRXN1, MECP2, SHANK3, FMR1, TSC1/2* and *UBE3A.*^78,79^ In addition, rare *de novo* mutations of *CHD8, SCN1A, SCN2A, SYNGAP1, ARID1B, GRIN2B, DSCAM, TBR1, KATNAL2, LAMC3* and *NTNG1* have been identified, with strong evidence for their association with ASD.^78,80–82^ Approximately 10% of them are copy number variations (CNVs) that disrupt protein coding, including chromosomal duplications, large deletions, inversions, and translocations, such as 1q21.1 duplications or deletions, 3q29 deletions, 7q11.23 duplications, 15q11-q13 deletions, 15q13.3 microdeletions, 15q11-13 duplications, 17q12 deletions, 22q11.2 deletions and 22q13.33 duplications or deletions.^78,83,84^ Mutations located in intronic and intergenic regions are the third variation type of ASD.^85^

ASD is thought to contain two subtypes: syndromic and non-syndromic forms. Syndromic generally refers to mutations in a specific gene or genome, manidesting as neurological syndromes (such as fragile X syndrome, tuberous sclerosis, Rett syndrome, Phelan–McDermid syndrome and Angelman syndrome).^79,85^ Non-syndromic, also regarded as idiopathic, which accounts for the vast majority, is not associated with other neurological disorders (or syndromes) but is related to some genes associated with autism.^85^ In heterogeneous genetic structures, syndromic ASD caused by high-penetrance single-gene mutations represent only a minority of ASD cases, the majority of cases are idiopathic.^86^ In fact, due to the overlap of phenotypes and growing understanding of intersecting biology, it remains controversial that the definition and boundary between syndromic and idiopathic ASD. With the advance of genetics, more efforts have been invested in identifying individuals with rare mutations of same gene and the convergence among them. Some retrospective analysis of gene fragments (for example, *CDH8* and *ADNP*) from individuals with typical idiopathic ASD has revealed different clinical phenotypic features.^87,88^ This suggests significant variability in the symptoms, as well as the persistence of previously overlooked syndromes in idiopathic ASD. Therefore, continuous and holistic analysis rather than isolated studies may help us better comprehend ASD.

## Neurobiological mechanisms of ASD

Due to the above unknown factors and challenges, many genetic variations associated with ASD have been suggested to be possibly concentrated on common molecular or cellular pathways. Key literature from recent years has suggested that ASD-associated genes enriched in aspect of transcription and translation, synapse, epigenetics, immunity and inflammation. These are closely related to the occurrence, development and outcome of autism. The first category is the dysregulation of important transcripts and translational signalling pathways.^15,89,90^ The second category involves synaptic proteins, including cell adhesion, scaffolding, and signalling molecules, which can affect synapse structure and function during different processes of synapse formation, elimination, transmission, and plasticity.^89,91,92^ The third category is the overtranslation of certain transcripts, which can lead to widespread epigenetic dysregulation, creating a positive feedback loop between translation and transcription processes that exacerbates neuronal dysfunction in ASD.^93^ The immunoinflammatory response caused by the activation of reactive glial cell proliferation and intestinal flora dysbiosis can be classified into the fourth type of abnormal signal transduction.^94,95^ These types of signalling pathways can interact or participate in the pathophysiology of ASD in a cascading manner rather than acting independently. For example, alterations in Wnt signalling, alterations in neuronal translation and defects in synaptogenesis or synaptic function during brain development can all affect the formation and activity of neural circuits.^96,97^ In turn, altered neural activity can further influence transcription factors or chromatin remodelling by transmitting action potential cascades that trigger signals and initiate specific transcriptional programmes.^89,98^

Numerous animal genetic models of autism have been developed and characterised as a result of genetic advances, allowing relevant phenotypes and mechanisms to be discovered and further studied (Table 1). Mouse models have provided a mountain of evidence for molecular pathways in autism, especially in translation and synaptic function.^15^ Manipulation of individual risk genes in model systems may lead to identification of important phenotypes. Although they cannot completely simulate the pathological process of human beings, these techniques still help us to understand the occurrence and development of autism. Stem cell models have also demonstrated that abnormalities in specific molecular processes contribute to the pathogenesis of ASD (Table 2), including chromatin remodelling, Ca^2+^ and Wnt signalling.^99,100^ In recent years, accumulated evidence from modelling studies has identified many specific types of viable mutations, which may paint a bright picture for elucidation of the underlying pathogenesis of ASD.

**Table 1:** Mouse models of ASD

Target	Mice	Behaviour phenotypes	Molecular, cellular and circuit phenotypes	Mechanism	Ref.
Nlgn	*Nlgn-*3 KO	Reduced ultrasound vocalization Impaired social novelty preference Olfactory deficit Increased repetitive behaviour	Selective synapse impairment	*Nlgn-3* mutations specifically impede synaptic inhibition on D1-dopamine receptor-expressing neurons	^370,557^
	*Nlgn-3* R451C	Impaired social interactions Enhanced spatial learning abilities	Altered inhibitory synaptic transmission Altered excitatory synaptic transmission Enhanced the complexity of dendritic branching	Neuroligin dysfunction altered the E/I balance and synaptic transmission	^193,195^
	*Nlgn-4* KO	Impaired social interactions and social memory Reduced ultrasound vocalization	Reduced brain volume	Loss of Nlgn-4 selectively impaired glycinergic synaptic transmission	^558,559^
Nrxn	*Nrxn-1α* KO	Increased repetitive grooming Deficient social behaviours Elevated anxiety Reduced nest building	Deficient excitatory synaptic strength Impaired PPI	Nrxn-1α deficiency reduced excitatory synaptic transmission and resulted in an E/I imbalance	^560,561^
	*Nrxn-2α* KO	Deficient social interaction Increased anxiety-like behaviour	Reduced spontaneous transmitter release at excitatory synapses in the neocortex Impaired NMDAR function	E/I imbalance	^562^
MeCP2	*MeCP2*^+/−^	Impaired motor coordination Increased anxiety Abnormal social behaviour Deficient contextual fear memory Breathing abnormalities	Reduced brain volume Enhanced PPI	Absence of MeCP2	^563^
	*MeCP2*-TG1	Motor defects Stereotypies and seizures Impaired social behaviour Anxiety-like behaviour	Increased *Crh* and *Oprm1* in the amygdala	Social approach deficits may be due to increased *Oprm1* levels	^564^
Shank3	*Shank3* e4–9 KO	Repetitive grooming Deficits in learning and memory Abnormal ultrasound vocalizations	Decreased levels of Homer1b/c, GKAP and GluA1 at the PSD Decreased NMDA/AMPA ratio at excitatory synapses Deficits in LTP	Homozygous deletion of exons 4-9 induce loss of isoforms of Shank3	^204,565^
	*Shank3B*^-/-^	Repetitive grooming Deficient social interaction	Altered PSD composition in the striatum Morphological defects of medium spiny neurons Reduced cortico-striatal synaptic transmission	Dysfunction of Nrxn/Nlgn/PSD95/SAPA-P/Shank complex	^202^
	*Shank3* HET	Impaired social behaviour Reduced ultrasound vocalization	Reduced basal neurotransmission	Shank3 deficiency influence AMPA receptor recruitment and synaptic development	^205^
	*Shank3*^+/ΔC^	Social deficits Repetitive behaviours	Diminished NMDAR synaptic function and synaptic distribution	Shank3 deficiency leads to the reduced expression of βPIX (GEF for Rac1), and Rac1/PAK/LIMK signalling	^566^
	InsG3680	Impaired social interaction Repetitive self-grooming Increased levels of anxiety Impaired motor coordination	Severe striatal synaptic defects Altered PSD composition Much minor molecular defects at cortical synapses at P14	Impaired synaptic transmission induced long-lasting alterations in striatal connectivity	^206^
Shank2	*Shank2*^−/−^	Repetitive grooming Abnormal vocal and social behaviours	Reduced dendritic spines basal synaptic transmission Decreased frequency of miniature excitatory postsynaptic currents enhanced NMDAR-mediated excitatory currents at the physiological level	Altered glutamatergic neurotransmission can lead to the core symptoms of ASD	^203,207^
	*L7-Shank2*^−/−^	Impaired motor learning Abnormal social and repetitive behaviour	Decreased AMPAR in cerebellar synaptosomes Increased sIPSCs and spiking irregularity Impaired synaptic and intrinsic plasticity in PC	*Shank2* deficiency impairs PC intrinsic plasticity and induction of LTP at the parallel fibre to PC synapse	^208^
Fmr1	*Fmr1* KO	Deficient social behaviour	Elevated basal protein synthesis LTD is exaggerated downstream of an mGluR5 signalling pathway	The absence of FMRP leads to enhanced activity of mGluR5 signal transduction pathways	^567,568^
Tsc	*Tsc1*^+/−^, *Tsc2*^+/−^	Deficient social interaction	Hyperactivation of mTOR	Uninhibited mTOR signalling pathways	^569^
	L7Cre; *Tsc1*^+/+^	Abnormal social interaction and vocalizations Increased repetitive behaviour	Decreased PC excitability	Overactivity of the mTOR signalling pathway	^146,376^
	*Tsc2*^+/−^	Deficient social interaction	Deficient spine pruning and cortical projection neurons Deficient autophagy	*Tsc2* mutations caused unregulated mTOR activity	^567^
Ube3a	*Ube3a* 1× and 2× transgenic	Defective social interaction Impaired communication Increased repetitive stereotypic behaviour	Suppressed glutamatergic synaptic transmission	Increased E3A ubiquitin ligase gene dosage results in reduced excitatory synaptic transmission	^570^
Chd8	*Chd8*^+/−^	Deficient social behaviour Communication difficulties Repetitive behaviour	Synaptic dysfunction within MSNs in the NAc Delayed neurodevelopment	Reduced expression of CHD8 is associated with abnormal activation of REST	^131,368^
Scn1	*Scn1a*^+/−^	Stereotyped behaviour Deficient social interaction Impaired context-dependent spatial memory	Decreased NMDAR synaptic function and synaptic distribution Decreased cortical actin filaments Insufficient NMDAR	*Scn1a* haploinsufficiency impaired GABAergic neurotransmission and NaV1.1 dysfunction induce behavioural and cognitive impairments	^181^
Syngap	*Syngap1* HET	Deficient social memory Tendency to social isolation	Dendritic spine synapses develop prematurely Premature spine maturation enhanced excitability	*SYNGAP1* deficiency impaired NMDAR-CAMKII-SynGAP-GluR1 pathway *SYNGAP1* haploinsufficiency altered E/I balance	^571,572^
Arid1b	*Arid1b*^+/−^	Abnormal cognitive and social behaviour	Decreased number of cortical GABAergic interneurons Reduced proliferation of interneuron progenitors in the ganglionic eminence Imbalance between excitatory and inhibitory synapses	*Arid1b* haploinsufficiency suppressed H3K9Ac overall, and reduced H3K9Ac of the Pvalb promoter, resulting in decreased transcription	^573^
Tbr1	*Tbr1*^+/−^	Impairment of social interaction, ultrasound vocalization, associative memory and cognitive flexibility	Defective axonal projections of amygdala neurons	*Tbr1* gene altered the expression of *Ntng1*, *Cntn2* and *Cdh8* and reduced both inter- and intra-amygdala connections	^110^
Pten	*Pten*^+/–^	Deficient social behaviour Repetitive behaviour Lower circadian activity Impaired emotional learning	Brain overgrowth Abnormal immune system Altered cytoarchitecture and synaptic	Desynchronized growth in key cell types	^574,575^
	Nse-cre; *Pten*^f/f^	Abnormal social interaction Heightened anxiety Decreased motor activity	Macrocephaly Neuronal hypertrophy Loss of neuronal polarity	Abnormal activation of the PI3K/AKT pathway in specific neuronal populations	^147,576,577^
	NS-*Pten* KO	Repetitive behaviour Deficient social behaviour	Decreased mGluR Increased phosphorylated fragile X mental retardation protein Decreased dendritic potassium channel Kv4.2 Decreased PSD-95 and SAP102	Hyperactivation of the PI3K/AKT/mTOR pathway	^578^
	Nestin-cre; *Pten*^f/f^	Impaired social interactions Increased seizure activity	Increased differentiation to the astrocytic lineage Stem/progenitor cells develop into hypertrophied neurons with abnormal polarity	Altered AKT/mTOR/GSK3β signalling pathway	^579^
En2	*En2*^-/-^	Deficient social behaviour Deficient novel object recognition memory and spatial learning Increased depression-like behaviour	Deficient PPI	*En2* deficiency influence SynI mRNA and protein levels	^580,581^
Cntnap2	*Cntnap2*^−/−^	Abnormal vocal communication Repetitive and restrictive behaviours Abnormal social interactions	Neuronal migration abnormalities Reduced number of interneurons Abnormal neuronal network activity Reduced cortical neuronal synchrony	*Cntnap2* deficiency may induce overactivation of direct pathway which promotes motor behaviour	^421^
15q11-13	*patDp*^/+^	Deficient social interaction Behavioural inflexibility Abnormal ultrasound vocalizations Correlates of anxiety	Increased [Ca^2+^]i response to 5-HT2cR signalling	Increased MBII52 snoRNA within the duplicated region, affecting 5-HT2cR	^582^
15q13.3	Df (h15q13)^/+^	Impairment in social interactions Restricted-repetitive behaviours Deficient communication	Enlarged brains and lateral ventricles Altered gamma-band EEG and ERPs	15q13.3 microdeletion impair expression of *Fan1, Mtmr10, Chrna7, Trpm1, Klf13*, or *Otud7a*	^583,584^
16p11.2	df/+ dp/+	Stereotypic motor behaviour	Increased numbers of Drd2 MSNs in the striatum Downregulation of DA signalling	16p11.2 deletion induce ENK dysregulation	^585,586^
22q11	Df (16)1/+	Deficient hippocampus-dependent spatial memory	Enhanced short- and long-term synaptic plasticity at hippocampal CA3–CA1 synapses Altered calcium kinetics in CA3 presynaptic terminals upregulated SERCA2	Presynaptic SERCA2 upregulation	^587^
_	(COX)-2^−^	Decreased motor activity Increased anxiety-linked behaviours Increased repetitive behaviours Deficient social behaviour	Altered expression of *Wnt2, Glo1, Grm5* and *Mmp9* Decreased glyoxalase 1 expression	Altered COX2/PGE2 pathway change neuronal cell behaviour and differential expression of genes and proteins related to ASD	^588^
_	mice treated with VPA	Decreased social interaction	Chronic activation of glial in the hippocampus and the cerebellum Increased expression of TNF-α and IL-6 in the cerebellum Increased microglia density in the hippocampus	VPA-treatment led to decreased expression of PTEN and increased levels of p-AKT protein	^297,589^
_	BTBR ^T+^ltpr3^tf^/J	Increased self-grooming Impaired social behaviour	Increased IgG and IgE in serum and IgG anti-brain antibodies Increased expression of cytokines in the brain Increased proportion of MHC-II-expressing microglia	Different autoimmune profile of BTBR mice is implicated in their aberrant behaviours	^298,590,591^
_	MIA	Deficient sociability Increased repetitive/stereotyped behaviour	Deficits in dendritic spine density, levels of synaptic proteins, synaptic transmission, LTP, and cortical malformations	Immune activation within the maternal compartment likely influences the developing fetal CNS through inflammatory mediators found in the blood and amniotic fluid of mothers	^37,286^

*Nlgn* neuroligin, *Nrxn* neurexin, *PPI* prepulse inhibition, *E/I* excitatory/inhibitory, *NMDAR* N-methyl-D-aspartate receptor, *PSD* postsynaptic density, *HET* heterozygous, *LTP* long-term potentiation, *PAK* p21-activated kinase, *LIMK* LIM-domain containing protein kinase, *sIPSC* spontaneous inhibitory postsynaptic currents, *AMPA* α-amino-3-hydroxy-5-methyl-4-isoxazole-propionic acid, P*C* Purkinje cell, *LTD* long-term synaptic depression, *REST* RE-1 silencing transcription factor, *mGluR5* metabotropic glutamate receptor 5, *ERPs* event-related potentials, *MSNs* medium spiny neurons, *SERCA2* sarco (endo) plasmic reticulum calcium-ATPase type 2, *COX2* cyclooxygenase-1, *PGE2* prostaglandin E2, *VPA* valproic acid, *MIA* maternal immune activation

**Table 2:** iPSC models of ASD

Target	Cell type	Molecular, cellular and circuit phenotypes	Mechanism	Targeting strategy	Ref.
NLGN4	Neurons	Fails to enhance synapse formation	ΔE4 mutation in NLGN4 compromises the ability of NLGN4 to induce synaptic differentiation	_	^592^
NRXN1α	Neurons	Increased sodium currents, higher AP amplitude and accelerated depolarization time Altered neuronal excitability and non-synaptic function Depressed calcium-signalling activity Impaired maturation of excitatory neurons	NRXN1α deletions can lead to neuronal hyper-excitability Deletion of NRXN1α lead to skewed differentiation of NES cells into immature and inhibitory neurons	_	^593,594^
MECP2	Neurons	Reduced synapses and spine density, smaller soma size Altered calcium signalling and deficient electrophysiological	Altered excitatory synaptic strength may underlie global network changes in RTT	IGF1 Gentamicin	^595^
	NPCs	Increased miR-199 and miR-214 Delayed GABA functional switch	miR-199 and miR-214 regulate extracellular signal-regulated kinase (ERK/MAPK) and protein kinase B (PKB/AKT) signalling Delayed GABA functional switch due to deficit in neuron-specific KCC2 expression	Overexpression mi-199 and miR-214 Restoring KCC2 level	^596,597^
	Astrocytes	Shorter total neurite length Decreased terminal ends	Loss of MeCP2 in astrocytes contributes to neuronal abnormalities MECP2 deficiency in neurons induces cell-autonomous dysfunctions	IGF-1 GPE	^598^
MECP2dup	Neurons	Increased synaptogenesis and dendritic complexity Altered neuronal network synchronization	MECP2 overexpression promotes early postnatal dendritic and synaptic growth	NCH-51 histone deacetylase inhibitor	^599^
SHANK3	Neurons	Altered morphologies of dendritic spines from pyramidal neurons Impaired both early stage of neuronal development and mature neuronal function Smaller cell bodies, more extensively branched neurites, reduced motility	Deficient excitatory synaptic transmission Lack of SHANK3 during early neuronal development may impair the structural integrity of neurons and lead to synaptic defects in later mature neurons	Rescued by transduction with a Shank3 expression construct	^600–602^
SHANK2	Neurons	Increased dendrite length, dendrite complexity, synapse number, and frequency of sEPSC	SHANK2 haploinsufficiency disrupts the complex interaction between synaptic formation and dendritic formation	Rescued by gene correction of an ASD SHANK2 mutation	^603^
FMR1	Neurons	Decreased expression of PSD95 Decreased synaptic puncta density, neurite length Higher amplitude and increased frequency of calcium transients Abolished homoeostatic synaptic plasticity	*FMR1* mutation induce functional differences in vGlut responses FMR1 inactivation impaired homoeostatic plasticity by blocking retinoic acid-mediated regulation of synaptic strength	Repairing the genetic mutation in the *FMR1* gene	^604,605^
	iPSCs	Altered cell fate commitment and cell cycle Cell-type-specific translational dysregulation Abnormal proliferation Increased protein synthesis	Hyperactive PI3K activity due to lack of FMRP may associated with deficient protein synthesis and proliferation	Inhibition of PI3K signalling	^606^
TSC2	NPCs	Increased proliferative activity and PAX6 expression Neurons differentiated showed abnormal morphology Increased saturation density and higher proliferative activity of astrocytes Slow differentiated into neurons	Enhanced mTOR pathway Reduced PI3K/AKT signalling and IRS1 expression	_	^607,608^
	Neurons	Increased cell body size and process outgrowth	mTORC1 hyperactivation	Rapamycin	^609^
UBE3A	Neurons	Impaired maturation of RMP and AP firing Decreased synaptic activity and synaptic plasticity	Changes in RMP may be directly related to UBE3A loss and AP and synaptic changes may be secondary effects	Pharmacologically unsilencing paternal *UBE3A* expression	^610^
CHD8^+/−^	Cortical organoids	Increased expression of *TCF4, DLX6-AS1* and *DLX1*	CHD8 affects GABAergic interneuron development, by modulating DLX gene expression	_	^611^
SYNGAP1	Neurons	Enhanced dendritic morphogenesis Stronger excitatory synapses and expressed synaptic activity earlier in development	SYNGAP1 regulates the postmitotic maturation of human neurons made from hiPSCs, which influences how activity develops within nascent neural networks	_	^612^
CDKL5 NTNG1	Neurons	Abnormal dendritic spines	CDKL5 contributes to correct dendritic spine structure and synapse activity CDKL5-dependent phosphorylation on S631 controls the association of NGL-1 with the postsynaptic molecular hub PSD95	_	^613^
RELN	NPCs	Decreased Reelin secretion Impaired Reelin–DAB1 signal transduction	Overactivation of the mTORC1 pathway contributes to the downregulation of the Reelin–DAB1 cascade	Rapamycin	^614^
CNTNAP2	Cortical organoids	Increase in volume and total cell number	Homozygous c.3709DelG mutation in *CNTNAP2* leads to abnormal brain development	Site-specific repair of c.3709DelG mutation using CRISPR-Cas	^615^
FOXG1	Neurons	Accelerated cell cycle Overproduction of GABAergic inhibitory neurons	Changed fate of GABAergic neurons induced by FOXG1	_	^616^
TRPC6	Neurons	Shortening of neurites Reduced dendritic spine density	MeCP2 levels affect *TRPC6* expression	TRPC6 complementation IGF1 Hyperforin	^617^
CACNA1C	Neurons	Deficient Ca^2+^ signalling Abnormal differentiation Abnormal expression of tyrosine hydroxylase Increased synthesis of norepinephrine and dopamine Activity-dependent dendrite retraction Abnormal migratory of interneurons	Ca(v)1.2 regulates the differentiation of cortical neurons in humans Ectopic activation of RhoA and inhibition by overexpressed channel-associated GTPase Gem	Roscovitine Pharmacologically manipulate LTCC	^108,618,619^
CNTN5 EHMT2	Neurons	Enhanced excitatory neuron synaptic activity	EHMT2 impacts the synaptic function of glutamatergic neurons through H3K9me1/2 catalyzing ability	_	^620^
15q11-q13	Neurons	Increased excitatory synaptic event frequency amplitude, density of dendritic protrusions, AP firing Decreased inhibitory synaptic transmission Impaired activity-dependent synaptic plasticity and homoeostatic synaptic scaling	Altered expression of UBE3A and other several genes in this region	Restoring normal UBE3A expression levels	^621,622^
15q13.3	Neurons	Increased endoplasmic reticulum stress Dysregulated neuronal gene expression Increased AP firing and elevated cholinergic activity Increased homomeric CHRNA7 channel activity	Common functional anomalies may be conferred by CHRNA7 duplication	Ryanodine receptor antagonist JTV-519 Wnt signalling agonist	^623^
16p11.2	Neurons	Increased soma size and dendrite length in 16pdel neurons Decreased neuronal size and dendrite length in 16pdup neurons Decreased synaptic density	Changes of the 16p11.2 region may influence genes encoding proteins that interact with the PI3K/AKT or Ras/MAPK pathway	_	^624^
22q11.2	Cortical organoids	Deficient spontaneous neuronal activity and calcium signalling Downregulated expression of miR-1290	Changed expression of DGCR8	Raclopride, Sulpiride, Olanzapine DGCR8 overexpression Overexpression miR-1290	^625,626^
22q13.3	Neurons	Reduced SHANK3 expression Deficient excitatory synaptic transmission	Loss of SHANK3	Restoring SHANK3 expression IGF-1	^28^
_	Neurons	Increased cell proliferation Abnormal neurogenesis Decreased synaptogenesis	Dysregulation of a β-catenin/BRN2 transcriptional cascade	IGF-1	^627^
_	Neurons	Decreased expression and protein levels of synaptic gene Decreased glutamate neurotransmitter release Reduced spontaneous firing rate	IL-6 secretion from astrocytes as a possible culprit for neural defects	Blocking IL-6 levels	^628^

*iPSC* induced pluripotent stem cell, *AP* action potential, *NES cells* neuroepithelial stem cells, *RTT* Rett syndrome, *IGF1* Insulin-like growth factor 1, *NPCs* neural precursor cells, *KCC2* K(+)-Cl(−) cotransporter2, *sEPSC* spontaneous excitatory postsynaptic currents, *PSD95* postsynaptic density 95, *vGLUT1* vesicular glutamate transporter 1, *RMP* resting membrane potential, *LTCC* L-type calcium channels, *IL-6* interleukin-6, *TS* Timothy syndrome

### Activity-dependent gene transcription and mRNA translation

**Fig. 3:**
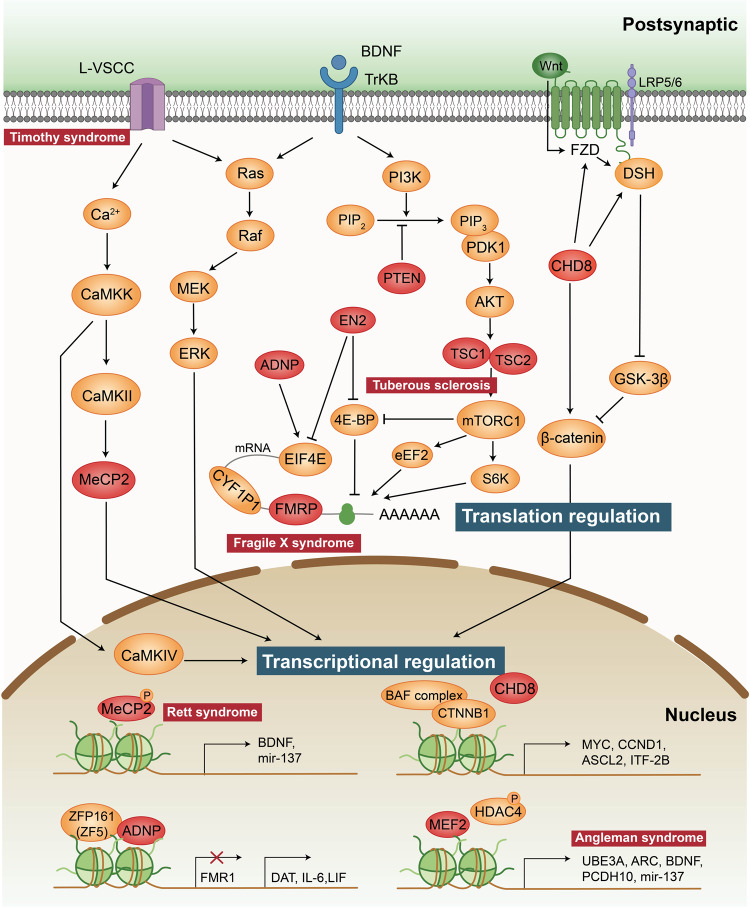
Transcription factors and translation mechanism associated with ASD. Activity-regulated translational pathways including the Ras/ERK and PI3K/mTOR. Both of them could be activated upon the stimulation of TrKB. Activation of L-type voltage-sensitive calcium channels (L-VSCCs) triggers calcium influx, induction of calcium-dependent signalling molecules and Ras/ERK pathways, involving in transcriptional regulation. These signalling cascades transcription regulators in the nucleus lead to the expression of transcription factors, thereby contributing to the regulation of activity-dependent gene transcription. Mutations of proteins involved in transcriptional regulation are associated with some syndromes of ASD, including L-VSCC in Timothy syndrome, MeCP2 in Rett syndrome and UBE3A in Angleman syndrome. Mutations of proteins involved in translation regulation including PTEN, ADNP, EN2, TSC1/TSC2 (tuberous sclerosis) and FMRP (fragile X syndrome). These genes have been highlighted in red

Neuronal activity regulates gene transcription and mRNA translation in a dynamic manner.^101–103^ Many transcription factors and *de novo* mutations associated with ASD are thought to regulate or engage in cross-talk with canonical Wnt signalling, such as *CHD8* and *CTNNB1*. Disorders in several upstream signalling pathways of translation, including mTOR, Ras and MAPK pathways, contribute to increased protein synthesis and therefore to altered synaptic plasticity (Fig. 3).

#### Activity-dependent gene transcription

Neuronal activity regulates programmes of gene expression in the nucleus, and disruption of activity-dependent transcriptional regulators or their targets is associated with ASD. Such disruption includes mutations in methyl-CpG-binding protein 2 (*MeCP2*),^104,105^ activity-dependent neuroprotective protein (*ADNP*),^106^ engrailed 2 (*EN2*),^107^ voltage-dependent calcium channel subunit α1C (*CACNA1C*),^108^ T-box brain 1 (*TBR1*),^109,110^ myocyte enhancer factor 2C (*MEF2C*)^111^ and de novo deletions or duplications in 15q11-q13 (which cover ubiquitin-protein ligase E3A (*UBE3A*)).^112^

*MeCP2* deletions or point mutations on the X chromosome in females manifest as Rett syndrome, a serious neurological disorder with autism-like symptoms.^104^ This is consistent with observations in model mice. Mecp2^308^/Y mutant mice exhibit ASD-like deficits in social behaviour and learning.^105,113^ MeCP2 is a transcriptional repressor which covers almost the whole genome, and its deletion raises overall transcriptional levels and accompanies with modification of the entire chromatin structure.^114,115^ Neuronal activity, brain-derived neurotrophic factor (BDNF), or drugs that increase intracellular 3’,5’-cyclic AMP (cAMP) levels induce MeCP2 phosphorylation and dissociation of the nuclear receptor corepressor (NCOR) complex, thereby enabling transcription.^116–118^ Notably, several studies have shown that MeCP2 binds with chromatin and transcriptional activators at the promoter of an activated target to activate gene expression, which means that MeCP2 can operate as both an activator and a repressor of transcription.^119,120^

Common genetic variations and rare mutations in genes encoding calcium channel subunits have extensive impact on the risk of ASD. For example, mutations in the L-type calcium channel Ca(v)1.2 generate Timothy syndrome, a monogenic disorder with a high penetrance for ASD.^108^ Transcriptional changes regulated by a series of calcium-dependent transcriptional regulators, including NFAT, MEF2, CREB, and FOXO, are found in Timothy syndrome.^99^ *ADNP* directly encodes a transcription factor and can bind and regulate ZFP161, which serves as a transcriptional activator of dopamine transporter (DAT; SLC6A3), interleukin 6 (IL-6), and leukaemia inhibitory factor (LIF) and a transcriptional repressor of FMR1.^121^ MEF2 is an activity-regulated transcription factor that regulates genes implicated in ASD, such as *ARC, PCDH10, UBE3A* and *BDNF.*^111,122,123^ The gene encoding the *UBE3A* is mutated in Angelman syndrome patients and duplicated on the maternal chromosome 15q11 in some ASD patients.^124^ Neuronal activity can promote the translation of *UBE3A* through the MEF2 complex.^125^ TBR1 is a neuron-specific transcription factor required for activity-dependent Grin2b expression, loss of a copy of which alters the expression of *Ntng1*, *Cntn2* and *Cdh8.*^109,110^

Notably, the majority of the targets of the above-discussed transcription factors also show crucial effects in synaptic transmission and plasticity, which may explain why transcription and translation can modulate synaptic function in the aetiology of ASD.^110,126–128^

#### Wnt signalling pathway

The Wnt signalling pathway has long been implicated in neuronal overgrowth, and its alterations are thought to be pleiotropic in the aetiology of autism.^129^ Molecular, cellular, electrophysiological, and behavioural abnormalities in accordance with autism-like phenotypes in several Wnt signalling-related knockout mouse models.^130,131^ In the brain, there are two primary pathways for Wnt signalling: (1) β-catenin-dependent stabilized “canonical” signalling and (2) β-catenin-independent “noncanonical” signalling.^96^ Notably, many key proteins in both signalling pathways are localized at synapses and play key roles in synaptic growth and maturation.^132–134^ Canonical Wnt signalling acts indirectly on β-catenin to enhance its stability, allowing it to translocate from the cell surface to the nucleus, thereby linking extracellular signalling to nuclear gene expression regulation through downstream transcriptional machinery (Fig. 3).^72^ On the one hand, ASD-associated MET tyrosine kinases (such as *CDH8*) release β-catenin to bind to surface calcium.^135^ On the other hand, free cytoplasmic β-catenin is phosphorylated by GSK3β to reflect the level of proteasomal degradation.^129^ Multiple Wnt molecules, including Wnt2, transmit signals at the surface membrane by interacting with frizzled receptors and LRP5/6 coreceptors.^136^

It is noteworthy that the gene *CTNNB1*, which encodes β-catenin, has been identified among ASD risk variation.^137^ *CDH8* is one of the best examples of an autism-related chromatin modifier that regulates the expression of other autism risk genes.^130,138^ As a negative regulator, CDH8 participates in the canonical Wnt signalling pathway by directly binding to β-catenin or being recruited to the promoter regions of β-catenin-responsive genes.^139^ This is consistent with the hypothesis that elevated canonical Wnt signalling contributes to the hyperproliferation of embryonic neural progenitor cells (NPCs) in the brain, which may partially explain the macrocephaly observed in individuals with autism.^88,100,140,141^ However, some studies have also found that CHD8 is a positive regulator of the Wnt/β-catenin signalling pathway in NPCs and negatively regulates this pathway in nonneuronal cell lines, suggesting that CHD8 may regulate Wnt signalling in a cell-specific manner.^130^

In addition, PTEN participates in Wnt signalling by working with β-catenin to regulate normal brain growth.^142^ A dynamic trajectory of brain overgrowth and elevated β-catenin signalling has been reported in the developing cerebral cortex in *Pten*-haploinsufficient mice, highlighting the roles of Pten and β-catenin signalling in regulating normal brain growth.^142^

#### Activity-dependent mRNA translation and protein synthesis

Several activity-regulated translational control pathways have been demonstrated to participate in pathologies of autism, such as the ERK/MAPK (mitogen-activated protein kinase)^143^ and PI3K/mTOR (mammalian target of rapamycin) pathways.^144,145^ Mutations in several genes, such as *TSC1, TSC2, PTEN* and *FMR1*, are canonical components involved in the mTOR pathways and play crucial roles in mRNA translation and protein synthesis.^146–148^

Tuberous sclerosis is an autosomal dominant disorder arising from heterozygous mutations in the *TSC1* and *TSC2* genes that is commonly associated with deficits in long-term and working memory, intellectual disability, and ASD.^22,149,150^ TSC1 acts as a regulator of the stability of TSC2, preventing the degradation of TSC2, while TSC2 is a GTPase activating protein (GAP) that inactivates Rheb, a GTPase of the Ras family, and other small G proteins.^151^ Activated AKT can phosphorylate and inhibit TSC2, which regulates translation, transcription, and other cellular processes by removing the inhibition of mTORC1 by the TSC1/2 complex and promoting mTORC1 activity.^151^ In the absence of a functioning TSC1/2 complex, overactive mTORC1 leads to unrepressed protein synthesis and subsequent cell growth.^152,153^ It is worth mentioning that a major activator of TSC1/2 signalling is BDNF, a secreted protein that binds to the receptor tyrosine factor TrKB and is thereby involved in the PI3K/mTOR pathway.^154,155^ *PTEN* is an ASD risk gene located on chromosome 10q23 that encodes a lipid specific for phosphatidylinositol (3,4,5)-triphosphate (PIP3), which is a negative regulator of PI3K/AKT/mTORC1 signalling upstream of TSC1/TSC2, resulting in symptoms of ASD. Mutations that inactivate *PTEN* lead to a constitutively active PI3K/AKT/mTOR signalling pathway and ultimately may result in abnormal protein synthesis.^156^

FMRP loss of function causes fragile X syndrome and autistic features, which is the most commonly known single-gene cause of ASD.^157^ FMRP is an RNA-binding protein whose target mRNAs encode transcription factors, and chromatin modifiers have been identified by high-throughput sequencing of RNA isolated with cross-linking immunoprecipitation (HITS-CLIP).^148,158–161^ The target genes of the mRNAs include several well-studied autism candidate genes, such as *ARC, NLGN3, NRXN1, SHANK3, PTEN, TSC2* and *NF1.*^23,148,162–165^ Notably, the proteins encoded by FMRP target mRNAs regulate the balance of activity-dependent translation in synaptic plasticity.^148^ The proteins include mGluR5 and the NMDAR subunits, consistent with findings of altered mGluR5 and NMDAR-dependent synaptic plasticity in fragile X syndrome mouse models.^166^ Moreover, mGluR activation regulates FMRP-mediated translational repression, whereas FMRP regulates AMPAR trafficking and mGluR-mediated LTD.^167^ Regarding the link between translation initiation and autism, FMRP interacts with cytoplasmic FMRP-interacting protein 1 (CYFIP1), which binds to the cap-binding protein eukaryotic initiation factor 4E (eIF4E) to form a protein complex that inhibits mRNA translation initiation and acts on the RAS-ERK pathway.^168,169^ Notably, the FMRP-eIF4E-CYFIP1 complex regulates the translation of more than 1000 genes, many of which are ASD risk genes.^170–173^ In addition, several transcriptional regulators, such as ADNP and ENP, also impact translation by interacting with eIF4E.^121,174^

In summary, current evidence suggests that there is a complex level of dynamic regulation between translation and transcription that likely contributes to ASD pathophysiology. Interestingly, most mutations in translation pathways such as mTOR, ERK, and FMRP-eIF4E-CYFIP lead to abnormally high levels of synaptic translation and synaptic proteins. This is one of the few convergences seen in the heterogeneous context of autism and provides a good foundation for pharmacological target development. Moreover, determining the dynamics of spatio-temporal relationship between transcription and translation will help us to link the molecular dysfunction to the complex behavioural characteristics of ASD patients.

### Synaptic function

**Fig. 4:**
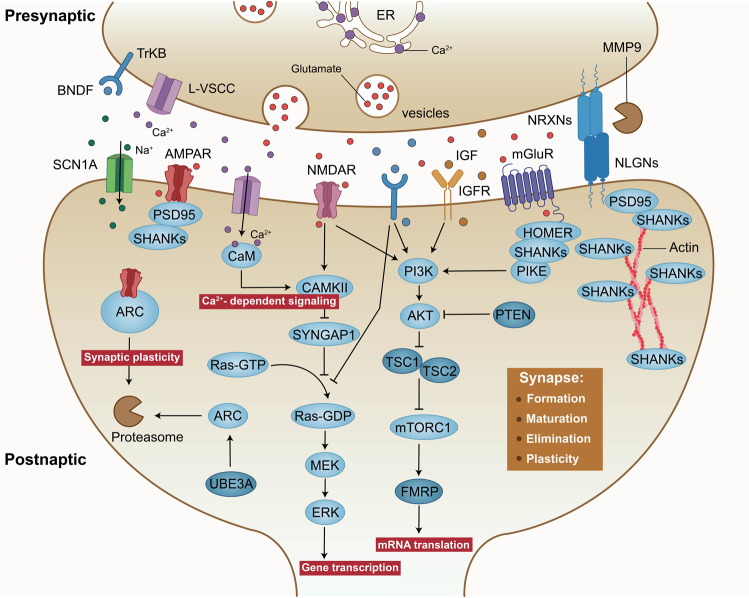
Molecular pathways implicated in synaptic function for ASD. At the excitatory synapse, encoded proteins including synaptic scaffold proteins (for example, SHANKs), neurotransmitter receptors (for example, NMDARs, AMPARs and mGluRs) and cell adhesion molecules (NRXNs and NLGNs) associated with autism risk genes. Activation of cell surface receptors is closely linked to activation of the Ras/ERK and PI3K/AKT/mTOR pathways. In addition, mutations in ion channels, such as L-VSCCs and sodium channel protein type 1 subunit-α (SCN1A), both of which have been illuminated result in synaptic dysfunction and autism-like behaviour

A growing number of genes that have been associated with ASD seem to play roles in synaptic structure and function by directly encoding synaptic scaffold proteins, neurotransmitter receptors, cell adhesion molecules, and actin cytoskeletal dynamics-related proteins (Fig. 4).^74,175^ Therefore, abnormalities in synaptic proteins might be some of the mechanisms that increase the risk of developing ASD. Among the synaptic proteins, cell adhesion molecules (neuroligins (NLGNs)^176^ and neurexins (NRXNs)^61^), postsynaptic scaffolding proteins (SH3 and multiple ankyrin repeat domains protein (SHANK),^177^ glutamate receptors (NMDAR subunit, GluN2B),^178^ inhibitory GABA_A_ receptor subunits α3 and β3 (GABRA3 and GABRB3, respectively)^179^ and permeable ion channels (voltage-dependent calcium channel subunit α1C (CACNA1C)^180^ and sodium channel protein type 1 subunit-α (SCN1A)^181^) are reported to be important signal transduction molecules associated with ASD. Signalling changes in these proteins can modulate the strength or number of synapses and ultimately alter the structure and functional connectivity of neuronal networks in the brain.

#### Synaptic structure and homoeostasis

Intact synaptic structure and homoeostasis are fundamental for the normal function of the brain. Neuropathological studies have provided evidence of increased dendritic spine density and aberrant dendritic spine morphology in individuals with ASD.^182,183^ Moreover, reduced developmental synaptic pruning in layer V pyramidal neurons in the postmortem ASD temporal lobe has been shown to hyperactive mTOR and defective autophagy.^146^ At excitatory synapses, the molecular diversity of surface receptors impacts proper synapse formation, maturation and transmission by organizing clustering of interaction partners at postsynaptic regions. For example, the intracellular carboxy-terminal portions of cell adhesion molecules (NLGNs) can bind to several scaffolding proteins of the postsynaptic density, such as postsynaptic density protein 95 (PSD95) and SHANKs.^184,185^ SHANK3 can interact with PSD95, AMPA receptor and glutamate receptor 1 (GluR1), which is critical for dendritic spine formation and synaptic transmission.^186,187^

NRXNs and NLGNs are presynaptic and postsynaptic binding partners that cooperate to form transsynaptic complexes that directly mediate synapse formation and stabilization but are abnormally manifested during autism pathology.^61,176,188^ Whereas NLGN-1, NLGN-3 and NLGN-4 localize to the glutamate postsynaptic membrane, NLGN-2 localizes primarily to GABA synapses.^189,190^ NLGNs can participate in the formation of glutamatergic and GABAergic synapses in an activity-dependent manner.^189^ Specifically, inhibition of NMDARs or the downstream protein CaMKII suppresses the formation of glutamatergic synapses through the activity of NLGN1, whereas inhibition of NLGN2 activity suppresses the formation of GABAergic synapses.^189,191,192^ Various combinations of these cell adhesion molecules have been linked to the differentiation of glutamatergic or GABAergic synapses in *Nlgn-3* and *Nlgn-4* mutant mice.^193–197^ In addition to alterations in NLGNs, mutations in NRXNs result in extensive changes in synaptic structure and plasticity.^198,199^ Moreover, NRXNs are critical for Ca^2+^-triggered neurotransmitter release but are not required for synapse formation, which has also been demonstrated in knockout mice.^198,199^

SHANK genes, including *SHANK1, SHANK2* and *SHANK3*, directly encode the proteins in the postsynaptic scaffolding protein family, which are located in the PSDs of excitatory synapses.^177^ SHANKs were first implicated in ASD by studies on Phelan–McDermid syndrome,^200,201^ a neurodevelopmental disorder caused by 22q13.3 deletion, and are deleted in almost all reported Phelan–McDermid syndrome cases. Consistent with studies in humans, different studies on *Shank* mutation sites in mice have also confirmed the strong genetic associations between *Shank* genes and ASD, especially *Shank3.*^202–208^ Individuals with ASD with *SHANK3* mutation exhibit defects in dendrite development and morphology and axonal growth cone motility.^209,210^ *Shank3*-knockout mice showed a decrease in the number of corticostriatal connections,^202,211^ whereas defects in NMDAR-dependent excitatory neurotransmission and synaptic plasticity have been observed in *Shank2*-knockout mice.^207^

In addition, recent genome-wide association studies have linked polymorphisms and rare variations in ion channels and their subunits to ASD susceptibility. Haploinsufficiency of *SCN1A* encoding the voltage-gated sodium channel Na(_V_)1.1 causes Dravet’s syndrome, which has been proven to result in the display of autism-like behaviour.^181^ The Na(_V_)1.1 channel is the major Na^+^ channel expressed in the somata and axon initiation segments of excitatory and inhibitory neurons in the brain.^212–214^ In GABAergic interneurons, Na currents and action potential firing are harmed when Na(_V_)1.1 is deleted.^181,215^ Calcium channels act as sensors electrical activity sensors, converting membrane potential changes into protein conformational changes and transmitting information about neuronal activity to downstream effector systems.

There is clear evidence to illuminate that defective Ca^2+^ channel function can lead to ASD with penetrance as high as 60-80%.^216^ Mutations relevant to ASD typically sensitize voltage-dependent Ca^2+^ channel gating, shifting their activation to more hyperpolarized potentials of ~10 mV.^217,218^ *CACNA1C* and *CACNA1D* encode the Ca(_V_)1.2 and Ca(_V_)1.3 proteins, respectively, which localize to the postsynaptic membrane and signal to the nucleus.^99,219^ In excitatory neurons, CaMKII functions as a shuttle molecule to collect Ca^2+^/Calmodulin from the cytoplasm and transport it to the nucleus, where Ca^2+^/Calmodulin release activates CaMKK and its substrate CaMKIV to further phosphorylate CREB, thereby participating in the regulation of transcription and translation.^72,220,221^

### Synaptic signalling pathways

Neuronal activity-dependent synaptic mRNA translation pathways can directly influence the levels of synaptic proteins, thereby controlling synaptic strength and number.^102^ The extracellular mTOR and FMRP-eIF4E-CYFIP1 signalling pathways are the two primary regulators of mRNA translation.^15^ Interestingly, the majority of ASD-related gene mutations (such as *MEF2C, FMR1, PTEN, TSC1, TSC2* mutations) result in enhanced gene transcription and mRNA translation, ultimately leading to an aberrant increase in the strength or number of synapses within certain neural networks. In fact, glutamate and BDNF can also induce a cascade of mTOR and FMRP pathways, resulting in an increase in mRNA translation.^74^ Consistently, increased glutamate and BDNF levels have been found in the blood of ASD patients.^222,223^

Moreover, activation of cell surface receptors such as NMDARs, AMPARs, mGluR, IGFR and TrKB is closely linked to activation of the ERK/MAPK and PI3K/mTOR pathways (Fig. 4). Among them, mGluRs are located in the perisynaptic zone of excitatory synapses, ideally contributing to orchestrating AMPARs and NMDARs.^224^ Mechanistically, mGluRs can directly regulate glutamatergic signalling by anchoring in complexes with SHANK and HOMER proteins and further control the synthesis of synaptic proteins via activation of the PI3K/AKT/mTOR pathways.^225^ In addition to being involved in dendritic protein synthesis, activation of mGluRs can also stimulate long-term depression (LTD), which is accompanied by rapid loss of both AMPA and NMDA receptors.^72^ Interestingly, several ASD animal models, including *Fmrp*-mutant,^167^ *Mecp2*-mutant,^113^ *Tsc1/2*-mutant,^226^ *Pten*-mutant,^227^ *Shank3*-knockout,^211,228^ *Nlgn3*-knockout^229^ and 16p11.2-knockout models,^26^ have shown dysregulation of mGluRs and abnormal mGluR-dependent LTD. There are encouraging signs that some pharmacological manipulations of mGluR have shown initial success in restoring impaired LTD and improving ASD-related behaviours in mouse models.^211,228^ These will be detailed in the section “THERAPEUTIC STRATEGIES”.

In addition, proteinases play posttranslational roles by regulating the activity-dependent cleavage of postsynaptic adhesion molecules at glutamatergic synapses. For example, the cleavage of NLGNs is triggered by NMDA receptor activation and is mediated by the proteolytic activity of matrix metalloprotease 9 (MMP9).^230^ The ubiquitin–proteasome system is required for the degradation of AMPA receptors, which influence synaptic elimination and plasticity.^231^ UBE3A modulates excitatory synapse development by regulating the degradation of ARC, which reduces LTP by promoting the internalization of AMPA receptors.^232^ Several studies have demonstrated that loss of function of UBE3A leads to increased ARC expression and subsequently decreases the number of AMPARs, ultimately impairing synaptic plasticity at excitatory synapses.^232,233^

### Epigenetic factors

**Fig. 5:**
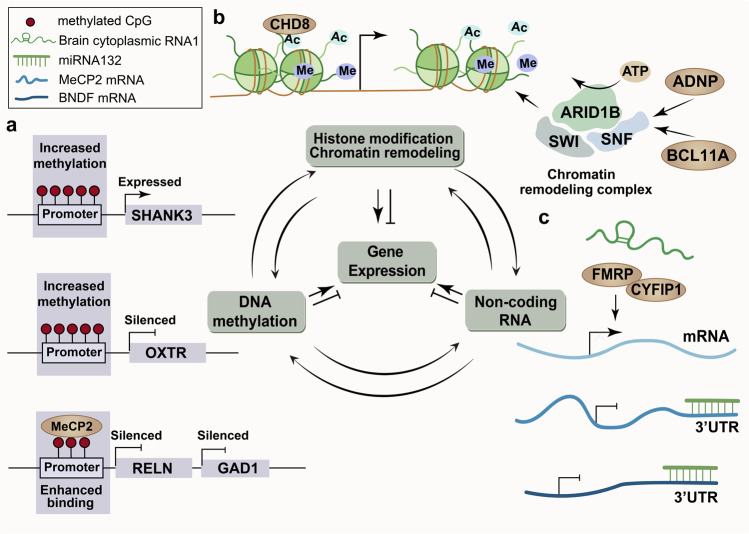
The epigenetic network associated with ASD pathophysiology. **a** Despite the exceptions, DNA methylation usually leads to transcriptional repression or even silencing of the affected gene. MeCP2 binds to methylated CpG sites in gene promoters and associates with chromatin silencing complexes, thereby suppressing gene expression. **b** Histone modification and chromatin remodelling cause transcriptional activation or inactivation, and chromatin packaging. **c** Non-coding RNAs control the expression of genes at the level of post-transcription by blocking protein synesis or inducing mRNA degradation

Increasing evidence indicates that ASD is the result of a complicated interaction between genes and the environment.^234^ Epigenetic factors are ideally positioned at the genome-environment interface, allowing for steady gene expression regulation without alterations to the underlying DNA sequence.^93,235,236^ Epigenetic mechanisms, including DNA methylation, histone modification, chromatin remodelling, and non-coding RNA activity, are involved in the regulation of social behaviour in autism.^93,237–239^ Together, these mechanisms form an epigenetic network that integrates transient social experiences into the genome to regulate social–emotional dispositions in mammals (Fig. 5).

#### DNA methylation

Many epigenetic researches have focused on DNA methylation with consideration of the contact between genes and environmental factors.^240–242^ Early studies on ASD-associated DNA methylation focused on several candidate genes, such as *MECP2*, glutamate decarboxylase 65 (*GAD65*), reelin (*RELN*), oxytocin receptor (*OXTR*), *SHANK3* and *UBE3A*.

MeCP2 is a chromatin architectural regulator and a reader of epigenetic information contained in methylation (or hydroxymethylated) DNA that has been well studied.^243^ Decreased MeCP2 expression in the PFC in ASD patients is associated with aberrant hypermethylation of its promoter.^244,245^ MeCP2 binds to methylated CpG sites in gene promoters and associates with chromatin silencing complexes, thereby suppressing gene expression.^246–248^ GAD1 and RELN are downregulated in postmortem ASD and are selectively expressed in GABAergic neurons.^249^ Enhanced binding of *MeCP2* to *GAD1* and *GAD2* promoters, which leads to reduced expression of RELN and mRNA, has been found in the cerebellum and frontal cortex in ASD patients.^249,250^ While the methylation rate of CpG islands is elevated during mouse brain development, SHANK3 is upregulated two weeks postnatal, suggesting that methylation of CpG islands is a strong regulator of SHANK3 expression.^251^ The neuropeptide oxytocin (peptide: OT, gene: OXT) sends signals via its receptor OXTR, which is a highly conserved G protein-coupled receptor. Both genetic and epigenetic changes in OXTR have been identified to be related to ASD.^252–255^ OXTR mRNA expression is affected by methylation of promoter, and high levels of methylation have been associated with ASD.^252,256^ Consistent with this, a study on siblings and adults with ASD found increased OXTR promoter methylation.^257,258^

Taken together, the findings indicate that DNA methylation status may serve as a potential biomarker for risk prediction, diagnosis, and targeting, as well as provide information for the study of ASD pathological mechanisms. Highly specific DNA methylation has been identified that may help predict transcriptional regulation in autism.^93^

#### Histone modification and chromatin remodelling

Recent studies have revealed a characteristic histone acetylation signature in the brains of ASD patients, providing strong evidence that histone modifications, especially acetylation, lead to ASD-like behaviours.^259^ A cross-generational study has confirmed that children exposed to prenatal anticonvulsants and the mood stabilizer valproate, a well-known histone deacetylase (HDAC) inhibitor, are at increased risk of being diagnosed with autism, providing insights into the involvement of histone modifications in ASD.^260,261^ Furthermore, treatment with a histone deacetylase inhibitor in *Shank3*-knockout mice significantly improves the behavioural phenotype of the mice, suggesting that abnormal histone modification is a potential mechanism of ASD.^262^ Trimethylation of the fourth lysine residue of histone H3 (H3K4me3) is essential for chromatin formation and gene activation, regulating hippocampal plasticity by recruiting chromatin remodellers to gene transcription initiation sites.^263,264^ H3K4me3-ChIP deep sequencing of the prefrontal cortex in postmortem tissue from patients aged 6 months to 70 years has revealed that alterations of H3K4me3 levels in neurons are associated with autism.^265^ Mutations in the lysine-specific demethylase 5 C (*KDM5C*) gene damage its function of transcriptional regulation, resulting in reduced H3K4me3 methyl group removal and suppressed gene expression in ASD patients.^266–268^

Chromatin remodelling is mediated via ATP-dependent enzymes or chromatin remodelling complexes.^269^ The chromatin structure or proteins that bind to DNA are altered when nucleosomes positioned differently, causing gene expression to shift. Chromatin remodelling genes (including *CHD8, ARID1B, BCL11A* and *ADNP*) have been identified to be linked to autism.^106^ De novo mutations in the autism-related chromatin modifier CHD8 are well studied,^88,270^ with multiple de novo, truncating, or missense mutations observed in ASD patients.^81,82,88,130^ CHD8 is located at active transcription sites with the histone modification H3K4me3 or H3K27ac and recruits histone H1 to target genes by remodelling the chromatin structure.^141,270^ *ARID1B* is a component of SWI/SNF (or BAF), an ATP-dependent human chromatin remodelling complex that is frequently mutated in ASD.^89,271^ Proteins encoded by *BCL11A* and *ADNP* can also interact directly with members of the SWI/SNF complex, which is related to alternative splicing of tau and prediction of tauopathy.^106,272^

#### Non-coding RNAs

The majority of genome-wide association studies have concentrated on protein-coding regions, disregarding non-coding RNA. Because non-coding RNAs primarily target transcripts and rarely interact directly with DNA, they are considered nonclassical epigenetic pathways.^93,273^ Posttranscriptional regulation by non-coding RNAs, including microRNAs (miRNAs) and long non-coding RNAs (lncRNAs), is associated with ASD. miRNAs are short non-coding RNA molecules that regulate the expression of most genes by blocking protein synthesis or increasing mRNA degradation at the posttranscriptional level. A preliminary assessment suggested that autism does not induce global dysfunction of miRNA expression, as only 28 of 466 miRNAs were significantly altered in postmortem cerebellar cortex tissue of ASD patients.^274^ Interestingly, the predicted targets of the differentially expressed miRNAs were enriched with genes related to neurobiology, the cell cycle, and cell signalling and largely overlapped with genes previously identified via differential mRNA expression analysis of ASD patients.^30,275^ Considering that miRNAs can be delivered into cells without being integrated into the host genome, miRNA-based therapy is a prospect strategy for the treatment of ASD.^237^ Highly expressed miRNAs in ASD patients can be downregulated by miRNA antagonist treatment (i.e., miRNA-inhibitory therapy), while miRNA mimic replacement therapy can compensate for weakly expressed miRNAs.^276^ Compared with mRNAs, lncRNAs exhibited higher tissue-specific expression, and a considerable number of lncRNAs were confined to the brain.^277^ The evolution of lncRNA-specific and synaptic function-enriched gene expression in primates suggests that this category of RNAs may have a broad range of roles in the brain and may help to elucidate the aetiology of ASD.^31,278,279^

In animal studies, mice with heterozygous knockout of miR-137 show repetitive behaviours and social behavioural deficits.^280^ Another example of the use of miRNA profile screens in a genetic model of ASD comes from a study on *Mecp2*-knockout mice. Expression profiling of miRNAs in the cerebella of *Mecp2*-knockout mice revealed the downregulation of a subset of miRNAs.^280^ Moreover, some of these miRNAs targeted BDNF, which is consistent with the finding that miR-132 targets MeCP2 and BDNF in vitro and is downregulated in the cortices of *Mecp2*-knockout mice.^281,282^ Therefore, the regulatory loop including BDNF, miR-132 and MeCP2 may be involved in ASD.^237,282^ The deletions in regions of differentially expressed lncRNAs are similar to those reported for miRNAs and mRNAs.^30^ BC1 is an lncRNA whose deletion in the mouse cortex can cause social dysfunction. The underlying mechanism is that BC1 tends to increase the affinity of FMRP and CYFIPI, both of which are ASD risk genes.^168,283,284^

In general, many differentially expressed and functionally significant non-coding RNA genes and overall epigenetic disorders have been identified in ASD patients and animal models. Preliminary evidence for a relationship between epigenetic regulation and social behaviour has been obtained at the animal level. Nevertheless, the epigenetic network is intricate, and the recently discovered genes with differential expression may be just the tip of the iceberg in the context of ASD. The important topic is how social stress induces temporary changes in the epigenetic network and whether gene expression might contribute to long-term social–behavioural adaptations. Future studies need to further identify more brain-specific epigenetic regulatory genes and clarify their practical functional significance.

### Immunology and neuroinflammation

**Fig. 6:**
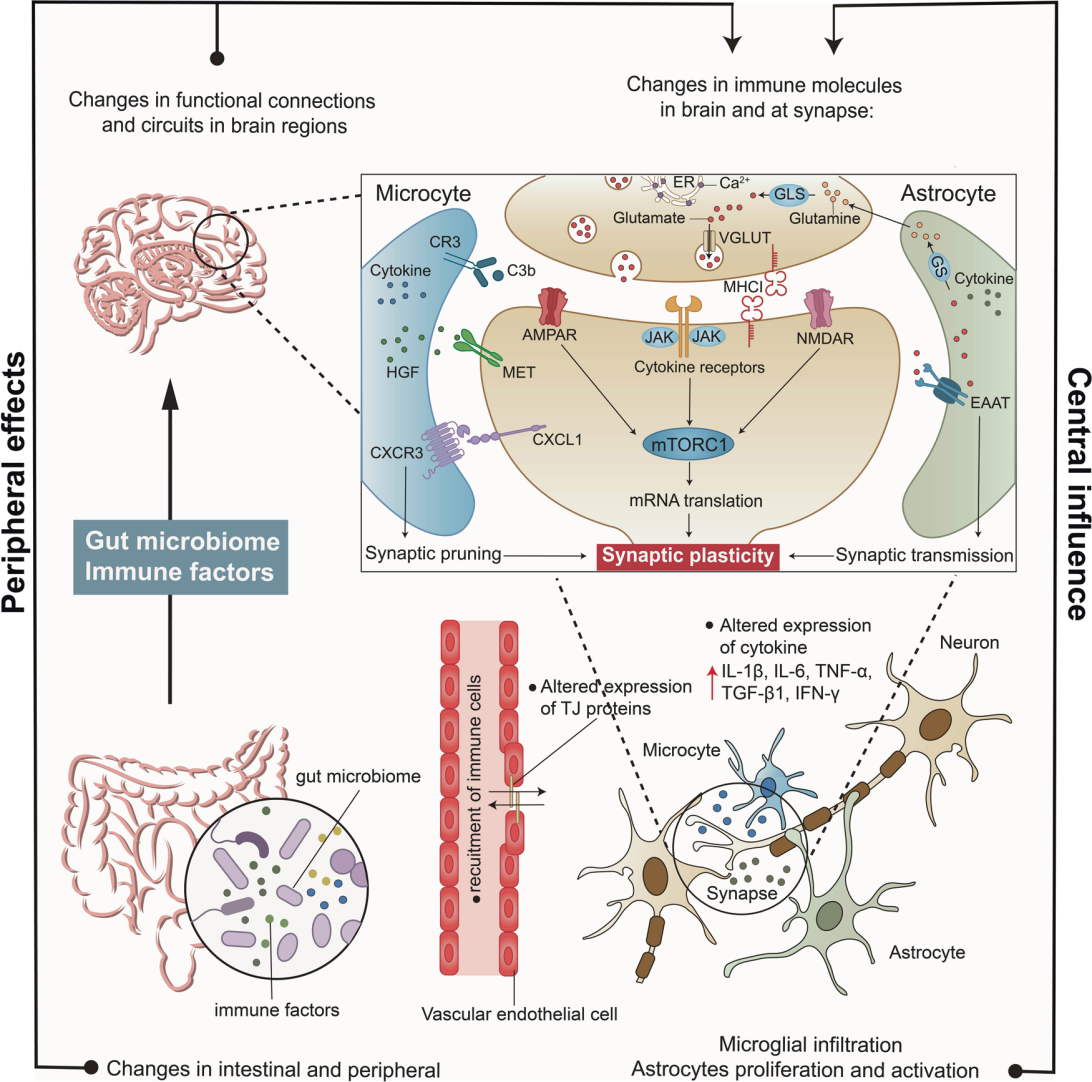
Mechanisms underlying the effects of microbiota, immunology and neuroinflammation on ASD. In periphery, microbiome and immune disorders in individuals with autism can lead to the change of peripheral immune environment. In the brain, abnormal proliferation and activation of glial cells can induce the secretion of cytokines and may cause vascular-endothelial dysfunction. Disorders in the periphery and brain all can affect brain functional connections and density of dendritic spines. Alterations in expression of immune mediators in the brain and at synapse, including cytokines and MHCI molecules. Notably, glutamate and cytokine receptors downstream signalling may converge upon the mTORC1 pathway, further regulating translation, synapse formation and plasticity. MHCI major histocompatibility complex class I molecules, mTORC1 mammalian target of rapamycin complex 1

Immune dysfunction is another factor attributed to gene–environment interactions in the context of ASD. Persistent immune dysregulation has been identified in ASD patients and animal models.^37,94,285,286^ An earlier study identified 150 differentially expressed genes in ASD patients compared to controls, 85% of which were upregulated and involved in immune response pathways.^275^ Inflammatory molecular signalling pathways in both the central nervous system and the periphery can affect brain connections and synaptic function by affecting components including microglia, complement factors, cytokines and their receptors, MET receptors, and major histocompatibility complex class I molecules (MHCI) (Fig. 6).^36^

#### Alterations of immune mediators in the central and periphery

In the brains of ASD patients, the numbers and activation of reactive microglia and astrocytes are increased in multiple brain regions.^30,287–291^ A cascade of cytokines and chemokines can be released by reactive microglia and astrocytes, which can signal across cells. Dysregulation of cytokines in ASD has also been associated with symptom severity and presentation on diagnostic tests for ASD.^292^ Therefore, abnormal cytokine profiles may be sensitive biomarkers indicative of immune system disturbances and abnormal neuroinflammation in autism. Some studies have found increases in GM-CSF, IL-6, IL-8, TNF-α, TGFβ, CCL2 and IFNγ levels in the brains of individuals with ASD, which supports this theory.^287,293^ Paralleling findings in humans, findings from several established animal models of ASD, including offspring with maternal immune activation (MIA) (IL2, IL6 and IL17)^294–296^ and offspring of VPA-treated rodents (TNF-α and IL-6),^297^ and the naturally occurring BTBR strain (IL-33, IL-18, IL-1β and CXCL7)^298,299^ have also shown alterations in the secretion of cytokines and chemokines. Due to the secretion of signalling molecules and cytokines, the cross-talk between microglia and astrocytes is enhanced, which can lead to vascular-endothelial dysfunction and damage to blood–brain barrier (BBB) permeability.^94,300^ Some cytokines, such as IL-1α, IL-1β, IL-6 and TNF-α, can migrate from the periphery into the brain via the BBB transport systems.^301^

Moreover, multiple studies have indicated different expressions of cytokine and chemokine in the periphery in autism patients.^94^ The results of cerebrospinal fluid and blood tests of ASD samples are similar, and cytokine changes in the blood can potentially provide information on inflammation and alterations in synapse connectivity in the brain. The levels of proinflammatory cytokines (such as IL-1β, IL-6, IL-8, IL-12p40, IFN-γ, TNF-α and GM-CSF) are increased, while those of anti-inflammatory cytokines (such as IL-10 and TGF-β) are decreased, in the blood of ASD patients.^302–304^ However, some alterations in cytokines are different between the central and peripheral regions, including IL-1β and TGF-β. In the CNS, IL-1β levels appear to be unchanged, but they have increased in the periphery.^293^ TGF-β1 levels have been reported to be rising in one study, while the vast majority of data point to a decline in TGF-β1 levels in peripheral blood.^287^ Hence, additional studies with persuasive datasets are warranted to confirm whether higher blood IL-1β levels influence CNS levels and whether TGF-β1 has dual roles in the brain and periphery in autism.

Notably, maternal autoimmune disorders, including autoimmune disorders (such as fever, allergies and asthma) and external exposures (such as mercury, lead, air pollutant, pesticide, and PCB exposures) can lead to elevated immune responses and increase ASD risk in offspring.^36,294,305,306^ The MIA model is an appropriate model for researching related mechanisms between maternal infection and ASD phenotypes. This model is created with influenza, viral infection molecules (poly(I:C)), bacterial mimics (*lipopolysaccharide*) and specific cytokines (such as IL-2 and IL-6).^37,38,307,308^ Poly(I:C) injection at midgestation generates offspring that display three core behavioural symptoms of ASD in all mice and some nonhuman primates.^37,309^ Changes in maternal cytokines such as IL-2, IL-6 and IL-10 levels, which may explain the MIA-induced ASD-like behaviours.^296,310^

#### Gut–brain axis of microbial–immune–neuronal communication

Recently, the gut gained attention as a key connection in the microbial–immune–neuronal system interplay. In addition to symptoms of inflammatory dysregulation, people with autism also experience gastrointestinal symptoms, including constipation, diarrhoea, and inflammatory bowel disease.^311,312^ The abundance of gut microbes in ASD patients, including *Clostridium*, *Desulfovibrio*, *Bifidobacterium* and *Bacteroides*, is significantly different from that in healthy controls.^313–317^ Consistently, several established animal models of ASD, including the naturally occurring BTBR strain (*Bifidobacterium* and *Blautia flora*), MIA model offspring (*Clostridium*),^318,319^ VPA-treated rodents (*Desulfovibrionales*)^320,321^ and mice lacking the synaptic adhesion protein SHANK3 (*Lactobacillus reuteri*),^322,323^ all show disturbance of the intestinal flora. Indeed, studies in animals and people with ASD have revealed that intestinal imbalance can affect peripheral immunological responses and contribute to immune cell dysfunction. For example, certain microbiota in the gut influence T-cell populations, and administration of *Bacteroides fragilis* restores the proper balance of T-cell populations in mice.^324^ Moreover, gut dysfunction affects brain function through neural, hormonal, and immune signalling.^95^ Interestingly, the gut microbiota is essential for microglial morphological and functional maturation, and microglial damage can be corrected to some extent by a complex microbiota.^325^ Therefore, microglia and inflammation alterations in the CNS may be at least partially attributable to microbial dysregulation.

#### Potential mechanisms of neuroimmune cross-talk

With the growing recognition and understanding of neuroimmune cross-talk, there is growing interest in how immune dysregulation affects brain functional connectivity. Most cytokines and their receptors are expressed by neurons and glial cells throughout development, and several studies have revealed that cytokines play important roles in neurogenesis, synapse formation, and plasticity, including IL-1β, IL-6, TNF-α, TGF-β1 and IFNγ^326–331^ Cytokines activate several signal transduction pathways, including the Janus kinase-signal transducer and activation of transcription (JAK-STAT) and PI3K/AKT/mTOR pathways, which regulate numerous cellular responses.^36,286,332^

In addition to participating in inflammatory responses, microglia and astrocytes also play key roles in maintaining brain homoeostasis by regulating synaptic morphology and plasticity.^333–336^ Specifically, glial cells engage in cross-talk with synapses through surface-expressed ion channels, receptors and transporters.^333–337^ Microglia regulate neuronal developmental remodelling and synaptic transmission by regulating the release of cytokines and chemokines in the adult brain.^334,336,338^ Consistently, significant impairments in synaptic pruning and synaptic transmission and ASD-like behaviours have been observed in CX3C chemokine receptor 1 (*Cx3cr1*)-knockout mice.^335,339^ These deficits may be attributable to increased signalling by IL-1β secreted from microglia.^339^ The engulfment of microglia is dependent upon the microglia-specific phagocytic signalling pathway via complement receptor 3 (CR3)/C3.^340^ This process is disrupted in mice with autism: increased C1q expression and enhanced phagocytic capacity have been found in the microcytes of *Pten*-mutant mice.^337^ Astrocytes affect synaptic transmission via glutamate uptake by the glutamate transporters GLAST and GLT1 and via regulation of synaptic function and plasticity mediated by calcium signalling.^341–344^ Correspondingly, astroglial GLT1 and glutamate uptake is significantly reduced in the cortex in *fmr1*^−/−^ mice, which may explain the enhanced neuronal excitability observed in mice with fragile X syndrome.^345^

On the other hand, immune molecules and their receptors, such as MET and MHCI, are involved in a wide range of physiological events during brain development.^36^ MET is an immune gene encoding hepatocyte growth factor (HGF), mutations in which induce disruption of multiple downstream targets in signalling cascades, resulting in critical functional deficits in brain development.^346,347^ Decreases in MET expression have been observed in ASD postmortem tissues.^348,349^ MET can indirectly lead to changes in neural circuits and functions by negatively regulating immune responses and gastrointestinal homoeostasis, which is a putative hallmark of ASD pathophysiology.^350,351^ In addition to mediating the adaptive and innate immune responses, MHCI molecules contribute to controlling axonal and synaptic growth and participate in the regulation of synaptic plasticity and synaptic homeostasis in the presynaptic and postsynaptic regions associated with glutamate.^352–356^ Cortical neurons from offspring of MIA exhibit increased expression of MHCI molecules and its downstream effect factors MEF2. Remarkably, normalizing the MHCI-MEF2 signalling pathway in cultured MIA neurons prevents the MIA-induced decrease in synapse density.^353^ Notably, despite recent advances, most of the details of when, where and how immune molecules function in the brain remain unknown.

In summary, dysregulation of immunoregulatory signalling molecules, including cytokines, microglial complement, MET, and MHCI, is an important link in the pathological process of ASD that possibly regulates synaptic morphology and plasticity in the CNS through common downstream pathways. Among them, mTOR serves as a focal point for integrating immunological signalling in the brain, cytokine signalling, perinatal environmental exposures, and chronic immune disorders. Determining whether and how immune contributions concentrate on the common mTOR pathway in future studies will be critical for our understanding of the importance of mTOR in different aspects, not just from an immune perspective, as well as for future targeted drug development.

## Brain functional connectivity and the neurotransmitter system

**Fig. 7:**
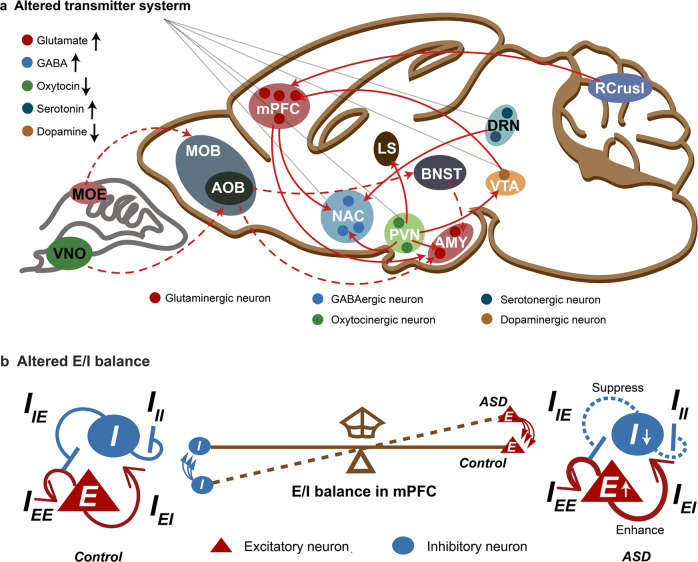
Social behaviour-related neural circuits, neurotransmitter system and E/I balance in the rodent brain associated with ASD. **a** A sagittal view of the rodent brain used to illustrate the local and distal circuits implicated in social behaviours. Recent studies use behavioural neuroscience, optogenetics, chemical genetics and electrophysiology have illuminated the relationships between various social behaviour and the activity of specific neural circuits. Alterations in brain connectivity usually accompany changes of neurotransmitter, including glutamate, GABA, oxytocin, serotonin and dopamine. **b** In addition, the hypothesis of disruption of cortical “E/I imbalance” in autism is widely accepted, which has also been highlighted in the figure. AMY amygdala, AOB olfactory bulb, BNST bed nucleus of the stria terminalis, DRN dorsal raphe nucleus, LS lateral septum, MOB main olfactory bulb, MOE main olfactory epithelium, NAc nucleus accumbens, PFC prefrontal cortex, PVN paraventricular nucleus, RCrusl right Crus I, VNO vomeronasal organ, VTA ventral tegmental area

Early brain development in people with ASD is accelerated, which leads to changes in brain connectivity, including physical and functional connectivity between different regions and concomitant neurotransmitter changes. Different types of genetic variants may disrupt the circuits of social interactions and repetitive behaviours, resulting in a complex matrix of genes, synapses, circuits, and behaviours. Here, we summarize and review these topics on three levels. We first describe abnormal functional connectivity in the brains of ASD patients at a macroscopic scale. We then summarize the results of recent animal studies at the level of neural circuits, providing insights into the mechanisms of multiple types of specific neuronal and molecular regulation of circuit networks (Fig. 7). Finally, we summarize the relevant signal transduction pathways that regulate neurotransmitters in ASD patients.

### Brain regions and neural circuits

According to human neuroimaging and neuropathological investigations, global brain developmental anomalies in children with ASD emerge in the cerebral cortex, striatum, cerebellum, brainstem, and other subcortical structures.^357–363^ Recent studies have identified that the medial prefrontal cortex (mPFC) integrates social and spatial information through neuronal coding. The mPFC is one of the best-studied brain regions related to social behaviour.^364,365^ In both mice and humans, several pieces of evidence imply that striatal dysfunction is a neurological substrate for repetitive behaviours.^366–368^ For example, *Nlgn1-*knockout mice exhibit ASD-like repetitive behaviours and corticostriatal synaptic abnormalities,^369^ whereas mice lacking *Nlgn3* exhibit similar behavioural changes caused by neuronal inhibitory transmission from D1-MSN in the nucleus accumbens (NAc).^370^ Mice lacking *Shank3* exhibit striatal hypertrophy and decreased corticostriatal excitatory synaptic transmission, as well as repetitive behaviours.^202^ In early assessments of autism, the amygdala exhibits reduced volume and increased neuronal density in the medial, central and lateral nuclei, which play critical roles in modulating fear conditioning, anxiety and social behaviour.^357,361,371–373^ Consistently, amygdalar axonal projections and neuronal activation are defective in Tbr1(+/−) mice, but these defects are ameliorated by infusion of an NMDA receptor agonist (D-cycloserine).^110^ The cerebellum is best known for its role in controlling motor behaviours, and most individuals with ASD have comorbidities associated with movement disorders such as ADHD. Histopathological changes in cerebellar neuronal structure, such as loss of Purkinje cells (PCs), have been discovered in the postmortem brains of many ASD patients.^357,374,375^ Validation data on key signalling molecules suggest that cerebellar PC-specific knockout of *Tsc1*, *Tsc2* and *Bmal1* is sufficient to induce core ASD-like behaviour.^376–378^ Notably, a growing number of studies have found that the cerebellum is involved in the pathophysiology of autism in the form of nonmotor regulation.^379–381^

Rodents and humans share similar brain regions and neural circuits, facilitating our investigation of social behaviour and related signalling mechanisms.^382^ Currently, rodents and nonhuman primates, such as chimpanzees, are accepted models for identifying social behavioural changes in autism. Numerous studies have shown that mice exhibit unique social behaviours, such as territorial aggression and mating, interpret olfactory traits as social information, and transmit and interpret emotional contagion and empathic responses.^383–385^ Novel approaches in optogenetics, chemical genetics, electrophysiology and behavioural neuroscience have helped to construct the links between various social behaviours and brain circuit activity (Fig. 7).^386–389^ In the huge and complex neural network involving social behaviour, the PFC and its massive reciprocal loop connections constitute a top-down social behaviour regulation system. Various subcortical networks communicate with the mPFC, including the amygdala (responsible for emotional processing), the NAc (responsible for social incentive), and the hypothalamus (responsible for stress regulation).^390–393^ Recently, the right crus I (RCrusI) of the cerebellum was identified as a key brain region for social interaction in mice that can project to the cortex to modulate social interaction and repetitive behaviours in mice.^394,395^ In addition, oxytocinergic, serotonergic and dopaminergic-related circuits also play critical roles in social regulation, which will be discussed below.

### Neurotransmitter system

From a neurobiochemical perspective, the activity of brain structures and neural circuits is coordinated by multiple neurotransmitters and neuromodulators. Therefore, dynamic changes in neurotransmitter concentration, release, and receptor density may directly affect neural circuit function and thus behavioural performance.^396^ Increasing evidence shows that disturbances in neurotransmitter systems, including the glutamate, GABA, serotonin (5-hydroxytryptamine, 5-HT),^397,398^ melatonin,^397,399^ dopamine (DA),^396,400,401^ OT and arginine vasopressin (AVP) systems, are associated with autism (Fig. 7).

#### Classic neurotransmitters. glutamate and GABA:

An appropriate balance between excitation and inhibition (E/I) in synaptic transmission and neural circuits is essential for appropriate brain functioning. In 2011, Yizhar et al. used optogenetics to study excitatory projection neurons and inhibitory PV neurons of the mPFC and subsequently found that an increase in the cellular E/I ratio leads to severe impairments in information processing and behaviour.^402^ Currently, the hypothesis of cortical “E/I imbalance” in autism is widely accepted (Fig. 7).^403–406^

E/I balance is controlled by the ratio of excitatory to inhibitory cells, as well as their activity. Plasma levels of GABA and glutamate are changed in autistic children, who exhibit significantly increased GABA levels and decreased glutamate/GABA ratios.^223^ Previous findings have highlighted the importance of glutamate dysfunction in contributing to the aetiology of autism.^407–411^ In addition to the above mentioned changes in glutamatergic neurons in ASD, the functional role of GABAergic inhibitory neurons is becoming increasingly clear. Neuropathological studies have provided evidence of reduced GABAR levels in the cortex and hippocampus, aberrant GAD1 and GAD2 mRNA expression in the postmortem cortex and cerebellum, and the interneuron markers parvalbumin (PV) and somatostatin (SST) are downregulated.^412–417^ Loss of inhibitory neurons and impairment of inhibitory neurotransmission are also observed in ASD mouse models as a result of mutations in genes such as *Pten, Mecp2, Cntnap2, Shank3* and BTBR mice, which may directly lead to alterations in the balance of excitation and inhibition.^418–423^ It is worth noting, however, that investigations on E/I imbalance have primarily been carried out using animal models, therefore a detailed assessment of the pathophysiology of E/I imbalance contributing to human ASD is warranted.

#### Biogenic amines. 5-HT and DA:

5-HT has long been suggested to be related to social behaviour. Early researches suggested increased 5-HT levels in the blood of children with autism. According to data from neuroimaging and neurobiochemical analyses, up to 45% of individuals with autism have hyperserotonaemia.^398^ Abnormal 5-HT neurotransmission and social behavioural deficits have been reported in SERT and MAOA mutant animal models.^398^ Serotonergic neurons are mainly located in the dorsal raphe nuclei (DRNs), which can project to the PVN of the hypothalamus and modulate OT release.^424^ Moreover, other brain areas, such as the NAc, can also receive projections from the DRNs and display OXTR. A study in mice has elucidated that the coordinated activity of OT and 5-HT inside the NAc is essential for social reward.^425^ These studies have highlighted the synergistic effects of 5-HT and OT in ASD.

The DA system is also involved in ASD, and an early study identified elevations in HVA (a DA metabolite) in the cerebrospinal fluid of patients.^426^ Children with autism have defects in mesolimbic dopaminergic signalling, such as decreased dopamine release in the prefrontal cortex and decreased NAc neural responses.^427,428^ The majority of DA-producing neurons are located in two primary regions, the substantia nigra (SN) and VTA, in the brain.^429^ VTA dopaminergic neurons project to various brain structures, such as the NAc, involved in the control of social cognition.^388,430^ Although DA release has long been linked to reward, there is growing evidence that DA is released in response to aversive behaviour.^431–434^ The NAc has been well studied for its role in reward processing behaviour, which is predominantly composed of inhibitory MSNs that differ in the type of DA receptor they express, D1R or D2R.^388^ Notably, the two subtypes of neurons may play different roles in social and repetitive behaviours.^435,436^

#### Neuropeptides. OT and AVP:

The neuropeptide hormones OT and AVP belong to the same superfamily, and genetic variants in *OXT*, *OXTR*, arginine vasopressin receptor 1a (*AVPR1a*) and *CD38* (lately demonstrated as essentiall for social behaviour because it mediates oxytocin secretion) have been verified to be associated with autism.^437–440^ Compared to neurotransmitters (approximately 5 ms), neuropeptides (approximately 20 min) display a substantially longer half-life and are stored in dense core vesicles, which are much larger in size and scope than synaptic vesicles.^441,442^ Hence, OT and AVP have much broader neuromodulatory roles and less spatial/temporal specificity than classical neurotransmitters.^442,443^ The changes in OT and AVP levels in autistic patients’ plasma are often associated with abnormal functional connectivity.^444^ For example, OT administration increases the connectivity of brain regions critical for processing socioemotional information, such as the NAc, amygdala and PFC.^445^ Studies in animals have implicated OT and AVP in mammalian sexual, territorial, attachment and social behaviours.^442^ Moreover, OT also plays a recognized role in anxiety, which is common a comorbid symptom of ASD.^446^

OT is mainly produced by neurons located in the paraventricular nucleus (PVN) and supraventricular nucleus (SON) of the hypothalamic–neurohypophysial system. Social cues induce OT release from the PVN; the OT acts on downstream structures such as the LS, amygdala, VTA and NAc.^425,447–449^ OT release from oxytocinergic neuron axon terminals in the VTA drives the excitability of dopaminergic neurons in the NAc, and eventual activation of the PVN–VTA circuit enhances social behaviour.^448^

For nearly two decades, an increasing number of studies on the modulation of circuits and neurotransmitter systems have gained insight into different brain areas and circuits involved in particular behavioural states. Nevertheless, it is unclear to what extent the mouse phenotypes recapitulate the relationships among neural circuits in autism. It should be noted that the human brain with its multimodal structure has undergone dramatic changes in brain regions such as the frontal and temporal lobes during evolution. Therefore, more comparative studies between primate and mouse models are required to precisely correlate neuroanatomical features with candidate brain circuits involved in ASD pathogenesis. More importantly, identification of molecular mechanisms that are specific to social behaviours and circuits is needed. Such information will be essential for developing targeted treatments aimed at ASD.

## Therapeutic strategies

**Fig. 8:**
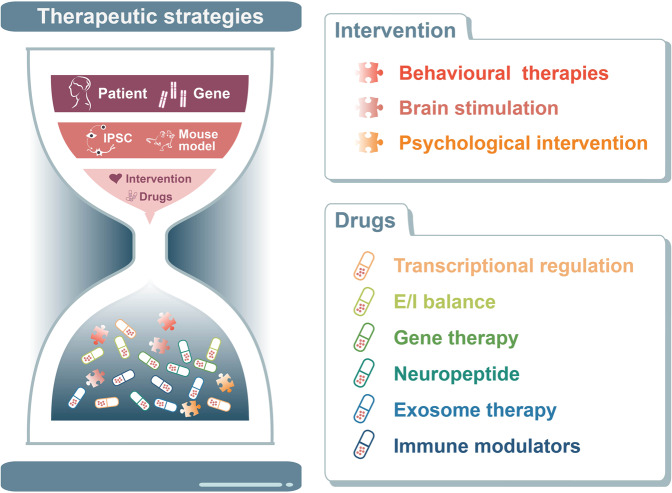
Potential novel therapeutic strategies and target of ASD. Abundant basic research on mouse and iPSC models exploited potential treatments to be used in ASD patients.It is noteworthy that emerging treatments including brain stimulation, gene therapy and exosome modulators are also been indicated

The current treatment strategies for autism are divided into nonpharmacological treatment and pharmacological treatment approaches. Combining pharmacotherapy with behavioural psychosocial learning interventions may have significant impacts on long-term outcomes for people with autism. However, based on the complex mechanism of the superposition of multiple aetiologies of autism, there is still a lack of clinical cures for core symptoms. In any case, the lack of molecular targets is the rate-limiting barrier for new drug research for autism. Innovative drug development for autism is currently the most challenging work in the field. The development of strategies to intervene in or block the transduction of key signalling molecules involved in the pathogenesis of autism is a primary research direction. In this section, we mainly review and discuss pharmacotherapies based on pathological features and signal transduction mechanisms (Fig. 8).

### Nonpharmacological therapies

Nonpharmacological treatment mainly refers to educational interventions and behaviour modification but also includes adjunctive treatments such as music and art therapy. The main purpose of nonpharmacological treatment is to develop children’s self-care and social skills, thus improving their quality of life. With advances in neuroscience, brain stimulation has also gradually attracted clinicians’ attention and has shown potential to improve the symptoms of ASD patients.^450,451^

#### Behavioural and psychological intervention

Physical intervention is usually considered as a priority because many young autistic children have difficulty communicating and interacting with others. Music therapy, cognitive behavioural therapy (CBT) and social behavioural therapy (SBT) have all showed promise in helping autistic patients improve their social interaction and verbal communication.^50,452^ One potential pathway by which music therapy affects ASD is by changing the structural and functional connectivity of the cortex to achieve a greater degree of multisensory integration across cortical and subcortical regeions during early development.^453^ CBT is a commonly used psychotherapeutic intervention and can both target core symptoms and treat comorbid anxiety and depression symptoms of ASD.^454,455^ SBT targets emotional regulation, social skills and functional communication, with an emphasis on independence and quality of life. Considering that the behavioural symptoms of ASD appear at a fairly early stage of development, intervening before symptoms appear may lead to better outcomes. Although treatments vary widely around the world, they generally follow a typical developmental psychology sequence that emphasizes play, social interaction, and communication with children. It is worth noting that clinical services should not be solely diagnosis oriented but should provide step-by-step specific interventions.^175^

#### Brain stimulation

Non-invasive brain stimulation is a relatively recent treatment option that has shown hope in the treatment of ASD. The molecular mechanisms underlying brain stimulation-dependent neuronal excitability and synaptic plasticity have been well elucidated with extensive preclinical animal models.^456–458^ Neuroimaging studies have demonstrated structural and functional imaging abnormalities in several brain regions of ASD patients. There have been more than a dozen trials of brain stimulation techniques, including transcranial direct current stimulation (tDCS) and transcranial magnetic stimulation (TMS), in the ASD population. tDCS is primarily conducted in the brain via a constant current through scalp electrodes. In contrast, in TMS, intracranial currents are induced in the cortex by fluctuating extracranial magnetic fields. Both techniques modulate regional cortical excitability and are well tolerated in children and adults.^459,460^ Neural stimulation has been reported to modify cortical excitability by affecting GABAergic function and causing LTP or LTD-like excitatory synaptic strength.^461–466^ tDCS has been shown to improve autism symptoms and language in several small clinical trials.^467,468^ Recent studies examining executive function in the dorsolateral prefrontal cortex (DLPFC) after TMS and improvements in social behaviour and social cognition in the posterior superior temporal sulcus and DLPFC in autistic patients after tDCS have shown preliminary therapeutic effects.^469–472^

Together, nonpharmacological therapies can partially alleviate autism symptoms. Although sufficient evidence is still lacking, the therapeutic effects of behavioural and psychological interventions and brain stimulation on autistic patients must have a theoretical basis related to neurobiochemistry and signal transduction.

### Drug targets and pharmacological therapies

Because the pathogenetic and pathological mechanisms are still unclear, there is no effective treatment drug for the eradication of autism that has been officially approved. Several drugs targeting autism are under study (Table 3) and clinical trials (Table 4). At present, clinical drug treatment of autism generally involves appropriate amounts of atypical antipsychotics, antidepressants, and sleep disorder-improving drugs according to the core symptoms of children.^50^

**Table 3:** Potential drugs under study

Drug	Pharmacological target	Improvement of symptoms	Clinical therapeutic effects	Adverse effects	Ref.
Guanfacine	Selective α2A adrenergic receptor agonist	Oppositional behaviour Anxiety Repetitive behaviour Sleep disturbance	Improved oppositional behaviour Significantly improved repetitive behaviour on the CYBOCS Effective in reducing oppositional behaviour Slightly improved repetitive behaviour	Drowsiness, fatigue, irritability decreased appetite	^478^
Melatonin	MT1R agonist	Sleep disorders	Effective in reducing insomnia symptoms	No serious AEs reported	^629^
Clonidine	α2-adrenergic receptor agonist	ASD relevant behaviour	Reducing sleep initiation latency and night awakening, slightly improve attention deficits hyperactivity, mood instability and aggressiveness	Sedation, dizziness or mild depression	^630^
Memantine	Non-competitive NMDAR antagonist	Social impairment	Significant improvement on the CGI-I and CGI-S	Increased seizures, irritability, emesis and sedation	^631^
		Language impairment ASD relevant behaviour Self-stimulatory behaviours	Significantly improve language function, social behaviour, and self-stimulatory behaviours	No serious AEs reported	^498^
		Cognitive, behavioural, and memory dysfunction	Significant improvement on CMSDLS and ABC subscales including hyperactivity, lethargy, and irritability Minimal improvement on CGI-I	No serious AEs reported	^499^
D-cycloserine	Partial agonist of NMDA glutamate receptor	ASD relevant behaviours	Significant improvement on the CGI and social withdrawal subscale of the ABC	Transient motor tic and increased echolalia	^632^
Baclofen	Selective GABA-B agonist	Irritability	Significant improvement for all the ABC subscales Greater effect on improvement of hyperactivity symptoms	No serious AEs reported	^633^
Arbaclofen	Selective GABA-B agonist	ASD relevant behaviours	Improvement on ABC-I, LSW, SRS, CY-BOCS-PDD, and CGI	Agitation and irritability	^509^
Bumetanide	Selective NKCC1 antagonist	Neurophysiological, cognitive, and behavioural measures	Significant improvement in irritable behaviour, social behaviour and hyperactive behaviour	No serious AEs reported	^512^
		Core symptoms of ASD	Significant improvement in symptom severity	Polyuria, mild hypokalemia, loss of appetite, fatigue, mild hyperuricemia	^513^
IGF-1	IGF-1R receptor agonist	Core deficits of ASD	Significant improvement in social impairment and restrictive behaviours	No serious AEs reported	^517^
Folate	Vitamin B	Language impairment	Improvements in subscales of the VABS, the ABC, the ASQ and the BASC for Children	No serious AEs reported	^526^
Oxytocin	Biological peptides	Repetitive behaviour Social deficits	Significantly reduce repetitive behaviours Improvements in affective speech comprehension from pre- to post-infusion	Mild side effects	^634,635^
Balovaptan	Vasopressin V1a receptor antagonist	Social behaviours	Improvements on the V-II ABC composite score	No serious AEs reported	^538^
Pioglitazone	PPAR-ϒ agonist	Core symptoms of ASD	Significant improvement in social withdrawal, repetitive behaviours, and externalizing behaviours	No serious AEs reported	^545^
PS128	Lactobacillus plantarum	ASD associated symptoms	Improved opposition/defiance behaviours Significantly improved in SNAP-IV	No serious AEs reported	^553^
MTT	Microbiota	Gut microbiota composition GI and ASD symptoms	Significant improvement in the GSRS, reduction of GI symptoms and significantly improved behavioural symptoms	No serious AEs reported	^546^
Paliperidone	Dopamine and serotonin receptors antagonist	Irritability	Improvement on the ABC-I	Mild-to-moderate extrapyramidal symptoms Weight gain	^636^
Donepezil	Cholinesterase inhibitor	ASD relevant behaviours	Significant improvement in ABC and the CGI-I Improvement in the Irritability and Hyperactivity subscales	Gastrointestinal disturbances Mild irritability	^637^
Mecamylamine	Nicotinic acetylcholine receptor	ASD relevant behaviours	Improvement in OACIS Decreased hyperactivity and irritability Improved verbalization	Constipation	^638^
Acamprosate	Modulate GABA transmission	Social impairment	Much improved on the CGI-I and improvement on both the ABC Social Withdrawal subscale and the total raw score of the SRS Improved hyperactivity as measured by the ABC Hyperactivity subscale	Reduced appetite Mild nausea	^639^
Amantadine	Noncompetitive NMDA antagonist	Hyperactivity Irritability	Significant improvements on ABC-CVs for hyperactivity and inappropriate speech Improvement on CGI	Insomnia	^640^
N-Acetylcysteine	Glutamatergic modulator	Behavioural disturbance	Significant improvements on ABC-Irritability subscale	No serious AEs reported	^641^
Olanzapine	5-HT2, DA receptor antagonist	ASD relevant behaviours	Significant improvement on three subscales of the ABC (Irritability, Hyperactivity, and Excessive Speech) and the TARGET	Weight gain, increased appetite, and loss of strength. extrapyramidal symptoms	^642^
Lurasidone	D2, 5-HT2A antagonist and 5HT1A partial agonist	Irritability	Significantly improvement in CGI-I	Vomiting and somnolence	^643^
Galantamine	Acetylcholinesterase inhibitor	Irritability	Improvement in ABC	No serious AEs reported	^644^

*ABC* Aberrant Behaviour Checklist, *AE* adverse effect, *CGI* Clinical Global Impressions (-I = Improvement, -S = Severity), *RFRLRS* Ritvo-Freeman Real Life Rating Scale, ABC-CV Aberrant Behaviour Checklist-Community Version, PDD pervasive developmental disorders, *CY-BOCS* Children’s Yale-Brown Obsessive Compulsive Scale, *CMSDLS* Children’s Memory Scale Dot Learning Subtest, *VABS* Vineland Adaptive Behaviour Scale, *ASQ* Autism Symptom Questionnaire, *BASC* Behavioural Assessment System for Children, *V-II ABC* Vineland-II Adaptive Behaviour Scales, *SNAP-IV* The Swanson, Nolan, and Pelham-IV-Taiwan version, *MTT* Microbiota Transfer Therapy, *GSRS* Gastrointestinal Symptom Rating Scale, *GI* gastrointestinal, *OACIS* Ohio Autism Clinical Impressions Scale, *SRS* Social Responsiveness Scale, *TARGET* a checklist of five target symptoms, Lethargy/Social Withdrawal subscales

**Table 4:** Potential drugs in clinical trials

Drug candidates	Pharmacological target	Improvement of symptoms	Registration number	Phase	Status	Ref.
Lurasidone	D2 and 5-HT-2A receptor antagonist	Irritability	NCT01911442	Phase 3	Completed	_
Atomoxetine	selective adrenergic uptake inhibitor	ADHD symptoms	NCT00498173	Phase 3	Completed	_
Paliperidone	D2 partial agonist and 5-HT-2A receptor antagonist	Aggression, self-injury, irritability	NCT00549562	Phase 3	Completed	_
Melatonin	MT1R agonist	Sleep disorders	NCT01906866	Phase 3	Completed	^645,646^
Oxytocin	Biological peptides	Social difficulties	NCT01944046	Phase 2	Completed	^647^
Guanfacine	Selective α2A adrenergic receptor agonist	PDD	NCT01238575	Phase 4	Completed	_
Acamprosate	GABA agonist and partial glutamate antagonist	Social skills deficits	NCT01813318	Phase 1	Completed	_
Memantine	Non-competitive NMDAR antagonist	Core symptoms of autism	NCT00872898	Phase 2	Completed	_
Nuedexta	NMDA receptor antagonist	Irritability	NCT01630811	Phase 2	Completed	_
D-cycloserine	Partial agonist of NMDA glutamate receptor	Symptoms of autism	NCT00198120	Phase 3	Completed	^648^
Arbaclofen	Selective GABA-B agonist	Social withdrawal	NCT01288716	Phase 2	Completed	_
Bumetanide	Selective NKCC1 antagonist	ASD	NCT03156153	Phase 2	Completed	_
Donepezil	Cholinesterase inhibitor	Communication skills, social interaction	NCT01887132	Phase 2	Completed	_
Mecamylamine	Nicotinic acetylcholine receptor	Core symptoms of autism	NCT00773812	Phase 1	Completed	_
Olanzapine	5-HT2, DA receptor antagonist	Disruptive behaviours	NCT00057408	Phase 2	Completed	_
Galantamine	Acetylcholinesterase inhibitor	ASD related	NCT00252603	Phase 3	Completed	_
N-Acetylcysteine	Glutamatergic modulator	Behavioural disturbance	NCT00627705	Phase 2	Completed	^641^
Pioglitazone	PPAR-ϒ agonist	Core symptoms of ASD	NCT01205282	Phase 2	Completed	^545^
Balovaptan	Vasopressin V1a receptor antagonist	Social behaviours	NCT01418963	Phase 1	Completed	^649^
		Socialisation and communication difficulties	NCT03504917	Phase 3	Completed	^650^
Amitriptyline	inhibition of serotonin and norepinephrine reuptake	Repetitive Behaviours	NCT04725383	Phase 3	Not yet recruiting	_
Mirtazapine	5-HT2 and 5-HT3 receptors antagonist	Anxiety	NCT01302964	Phase 3	Completed	^651^
Tasimelteon	Melatonin receptor agonist	Sleep disturbances	NCT05361707	Phase 3	Recruiting	_
IGF-1	IGF-1R receptor agonist	Social withdrawal	NCT01970345	Phase 2	Recruiting	_
JNJ-42165279	Fatty acid amide hydrolase	Symptoms of autism	NCT03664232	Phase 2	Recruiting	_

Atypical antipsychotics, including risperidone (a dopamine antagonist) and aripiprazole (a dopamine agonist), are FDA-approved drugs that have been shown to relieve irritability symptoms such as aggression and self-mutilation in adolescent autistic patients in several large clinical trials.^473–477^ α-Adrenergic drugs such as guanfacine are used for ADHD and disruptive behaviour.^478^ Antidepressants such as SSRIs improve the symptoms of emotional instability, anxiety, and stereotyped repetitive behaviours in patients with ASD by blocking the reuptake of 5-HT and increasing the concentration of 5-HT in the synaptic cleft.^479^ Fluoxetine, sertraline, citalopram, escitalopram, and fluvoxamine are SSRIs widely used in ASD. However, SSRIs are not suitable for everyone and should be used with caution, especially in people with autism with anxiety or obsessive–compulsive disorder.^480^

Notably, nearly 40–86% of children with autism have sleep–wake rhythm disturbances.^481,482^ Clinical drugs that can treat ASD by improving sleep include melatonin, ramelteon, niperrazine, and clonidine.^483,484^ It is worth mentioning that many investigations have reported aberrant melatonin secretion in autistic patients, particularly decreased melatonin and metabolite secretion at night, and altered circadian rhythms of melatonin.^481,484,485^ Several clinical trials have shown that melatonin reduces sleep latency and improves sleep duration and nighttime arousal, suggesting that it is an effective treatment for sleep disturbances in children with ASD. In addition, a meta-analysis and some placebo-controlled studies have suggested that melatonin supplementation may also have positive effects on autistic behavioural disorders.^481,486^ One study on VPA-treated rats has proven that melatonin treatment significantly improves social behavioural deficits through CaMKII/PKA/PKC signalling.^487,488^ Therefore, melatonin or novel analogues may be promising drug therapies for improving behavioural disorders in autism. In the future, it will be necessary to study the regulatory mechanism of melatonin-related signal transduction and to verify the dose–response relationship in the improvement of behavioural disorders in clinical trials to test the therapeutic benefits of melatonin.

In addition, the development of other ASD-targeted drugs has been promoted due to in-depth basic scientific research on the pathogenesis of ASD in the past decade. Clinical trials targeting E/I balance, transcriptional and epigenetic regulation, immune regulation, biological peptides and intestinal flora are advancing in an orderly manner (Table 3).

#### Targeting E/I balance

The cortical E/I imbalance hypothesis in ASD patients highlights the potential of glutamate and GABAergic receptor modulators as therapeutic agents.^402^ Different pharmacological methods have been applied to restore E/I imbalance, such as mGluR5 antagonist treatment, NMDAR agonist treatment and GABAR agonist treatment.^489–491^ Extensive preclinical data demonstrate that overactivity of mGluR5 is central to the pathogenesis of fragile X syndrome.^25,211,492^ In addition to targeting fragile X syndrome, mGluR5 inhibition has been shown to salvage many phenotypes, including learning and memory deficits, social deficits, repetitive behaviours, hyperactivity, and dendritic spine dysmorphogenesis, in 16p11.2 deletion mice, BTBR mice and *Shank3-*knockout mice.^26,228,493^ Unfortunately, mGluR5 inhibitors developed by two companies have exhibited negative effects in large-scale patient trials targeting fragile X syndrome.^494,495^ Further reasons should be sought for the discrepancies in preclinical and clinical outcomes. In addition to expanding and refining the preclinical analyses of new drugs, it will also be necessary to scientifically stratify patients enrolled in clinical trials in order to increase the expected efficacy in patients.

NMDA receptors and mGluRs show positive reciprocal regulation. NMDA receptor agonist (d-cycloserine) intervention attenuates impaired sociability in *Shank2*-transgenic mice, highlighting the need for accurate signalling at excitatory synapses.^207^ The spatial and temporal selectivity offered by subtype-selective positive allosteric modulators of the NR2 receptor make these agents promising candidates for the treatment of ASD.^496^ Drugs targeting the NMDA receptor, such as memantine, have been demonstrated to alleviate core symptoms of ASD in early open-label trials.^497–500^ Although subsequent RCTs have shown no differences in primary and secondary indicators, memantine improves symptoms of ASD such as stereotyped behaviours, and social communication/interaction impairment as an adjuvant therapy.^501–503^ The results from the memantine trial have been mixed, suggesting that further research is needed, and a large randomized controlled trial is currently being conducted on the therapy of social impairment in adolescents. Several trials on other NMDA-modulating drugs, including ketamine,^504^ riluzole,^44,505^ and d-cycloserine,^506^ have been negative for the primary endpoint, indicating that further studies with increased sample sizes are required.^506^

Evidence from fragile X syndrome mice has indicated that alterations in GABA-mediated synaptic transmission are present in the mice, suggesting that there is potential therapeutic benefit of GABA receptor agonism.^423^ Arbaclofen, a GABA-B agonist, regulates glutamatergic activity through presynaptic action to reduce glutamate release. In *Fmr1*-knockout mice, arbaclofen reverses protein synthesis, synaptic abnormalities and dendritic spine density phenotypes.^507^ Consistently, two clinical studies have suggested that arbaclofen has the potential to improve symptoms of ASD.^508,509^ Bumetanide, an NKCC1 (Na^+^-K^+^-2Cl^−^ cotransporter) chloride-importer inhibitor that reduces (Cl^−^)_i_ levels, enhances GABAergic inhibition, which improves the behavioural symptoms of individuals with ASD.^510–512^ Data from three follow-up studies have been obtained: two studies showed improvement in the primary endpoint (the Childhood Autism Rating Scale),^513^ while the other study showed no difference in the primary endpoint (the Social Responsiveness Rating Scale).^514^

#### Targeting translation and epigenetic regulation

Transcriptional and translational studies have provided a scientific foundation for the discovery of drug targets for underlying mechanisms, such as PI3K/mTOR pathways.^491^ mTOR inhibitors, such as rapamycin and everolimus, have been utilized to cure behavioural and molecular abnormalities in TSC-deficient mice.^22^ Unfortunately, chemotherapeutic agents acting on the mTOR pathway have not been discovered to improve social interaction of children with tuberous sclerosis.^515^ Preliminary data have shown that the pharmacological effects of IGF-1 affect synaptic development primarily by modulating the MAPK and mTOR pathways, as validated in Phelan–McDermid syndrome and Rett syndrome.^28,29,516^ Specifically, IGF-1 treatment results in increases in synaptic protein levels and activation of signalling pathway proteins and enhances cortical excitatory synaptic transmission and dendritic spine density. Trials of the effects of IGF-1 on social interactions in individuals with ASD have shown positive results, but larger trials will provide more definitive information on efficacy.^517–519^

In terms of epigenetic regulation, many autism risk genes are involved in histone modification and chromatin remodelling, and disruption of this process has been observed in individuals with autism. Treatment strategies with epigenetic enzymes, primarily targeting histone modifiers (such as histone deacetylase,^520^ histone demethylase^521^ and histone methyltransferase,^522^) show therapeutic potential in animal models. The Shank3-mutant mouse model is one of the most commonly used models to study epigenetic enzymes, and it was found that using histone methyltransferase inhibitors and histone acetylase inhibitors alone^520–522^ or in combination^523^ can both significantly improve NMDA dysfunction and social interactions in Shank3-mutant mice. In a recent small randomized controlled trial, dietary supplementation with methylation-modifying leucovorin/folate improved core symptoms of ASD.^524^ Folate is crucial to normal neurodevelopment. Abnormal folate metabolism has been identified in patients with ASD.^525^ Three randomized double-blind placebo-controlled trials evaluated the effect of folic acid on verbal communication in patients with ASD.^524,526,527^ Encouragingly, compared to placebo, folic acid improved scores in communication and social interaction, providing promising preliminary evidence for language impairment in children with autism.

#### Other biological targets: biological peptides, neuroinflammation and the intestinal flora

The neuropeptide theory of autism is backed up by evidence from animal research.^528,529^ OT has been discovered to play an important role in relationship formation and social functioning.^530^ Dozens of clinical trials have studied the effects of intranasal oxytocin on ASD.^531–534^ Although there is no substantial treatment-specific improvement in core social symptoms, recent findings on the long-term beneficial effects on repeated behaviours and feelings of avoidance are encouraging and suggest that OT may have therapeutic promise in the treatment of ASD. Given the difficulty of exogenous drug interventions in penetrating the blood–brain barrier, several trials on strategies to promote endogenous OT production are underway. AVP is a neuropeptide primarily used to regulate renal water reabsorption and increase perivascular resistance that has been detected at lower levels in the cerebrospinal fluid of ASD children than in controls and has also been studied as a target for ASD drug therapy.^535,536^ A randomized double-blind controlled trial of intranasal AVP in children showed a beneficial effect on sociability deficits.^537^ Combined with evidence from preclinical studies, this evidence indicates that V1a receptor antagonists may exert prosocial, antidepressant, and anxiolytic effects in disorders of social and emotional dysfunction. In a large trial conducted in adult men, balovaptan, an orally administered selective vasopressin V1a receptor antagonist, showed promise in terms of improving social interaction and communication among people with ASD.^538^

Findings of elevated levels of inflammatory factors and altered gut bacterial stages in children with ASD underscore the importance of ASD immune mechanisms.^539–542^ Peroxisome proliferator-activated receptor (PPAR-ϒ) is a nuclear hormone receptor, and its anti-inflammatory function has received attention. Pioglitazone belongs to the thiazolidinediones drug class (TZDs) and acts on PPAR-ϒ. In addition, pioglitazone has been identified to reduce NMDA-mediated Ca^2+^ currents and transients.^543^ Two clinical trials have suggested that pioglitazone has the potential to improve behavioural symptoms of ASD.^544,545^ Basic and clinical data have emphasized the role of gut microbes in the regulation of brain immune function.^546^ Modulating the microbiome has been shown to improve social core symptoms and synaptic dysfunction in animal models.^322,547–549^ Clinical trials have demonstrated that children with ASD treated with microbiota transfer have significantly reduced abdominal pain, indigestion, diarrhoea and constipation. In addition, the abundance of *Bifidobacterium, Prevotella* and *Desulfovibrio* is significantly increased, and the increases are correlated with improved symptoms.^546,550–552^ A recent study has also shown that *Lactobacillus plantarum* intervention in children with ASD reduces common abnormal behaviours and social impairments in ASD patients.^553^ Multimodal interventions are aimed at achieving clinical maximal therapeutic effects. It is expected that drugs targeting specific facets of autism will be developed to improve the core symptoms of patients. New drugs that affect synaptic plasticity, social learning or neuroinflammation must be combined with psychological interventions to achieve complementary synergies that ultimately have a major impact on the long-term outcomes of individuals with autism.

## Conclusion and perspectives

In conclusion, ASD is a complex disease caused by a series of combinations of different aetiological factors, including genetic factors, environmental and immune activation, etc., and ultimately manifests as abnormal changes in molecular signalling pathways, neuronal synapses, immune environment and brain functional connections. Animal models provide an opportunity to identify potential changes in circuit levels and their relation to behaviour regulation. Frustratingly, present medication only target concomitant symptoms rather than the core symptoms of autism, and the development of key molecular targets for signal transduction pathways is still in the basic research. To date, few trials have reached their primary endpoints, and little evidence has promoted the approval of drug administration agencies or the use of the tested treatments in clinical practice. For example, the efficacy of several small molecular targets has been well demonstrated in animal models, such as mGluR5 inhibitors, OT, Memantine, and mTOR inhibitors, but is still unsatisfactory in clinical trials. A serious challenge is how ASD can bridge the vast gap between molecular, cellular, and circuit convergence mechanisms to the heterogeneity of clinical manifestations. Therefore, basic research to clinical transformation remains the rate-limiting step in the development of treatment strategies for ASD, and the degree of heterogeneity may be considered, which may obscure the effect of experimental treatments. Conducting in-depth mechanistic studies using models such as nonhuman primates that can truly simulate human pathological processes would be crucial. The development of methods for manipulating nonhuman primate genomes may provide key insights for translation from model system experiments to human studies.

Despite these challenges, new therapies based on elucidated genes have been developed in recent years, such as gene replacement, gene editing and translating oligonucleotides.^554^ Relatively modest manipulation of gene expression using normal alleles may be sufficient to mitigate the effects of deleterious mutations. The development of technologies such as CRISPR–Cas9, which is based on targeted DNA editing, has facilitated rapid progress in gene therapy, and these technologies have also shown therapeutic effects in mice with fragile X syndrome. Thus, gene editing provides a new personalized medicine approach for the treatment of autism.^555,556^

To optimize and change the treatment strategy for autism, it is necessary to bridge biochemical molecular events, electrical oscillations and information processing and to explore the pathological mechanism of autism from a new systemic perspective. The coexistence of many clinical disorders in autism is quite common, but this autism comorbidity has not received enough attention thus far. Studies exploring potential biomarkers should design laboratory tests related to specific clinical syndromes based on the presence or absence of some specific comorbidities. Such research will require large-scale clinical cohort studies involving the same population, as well as focusing on spatiotemporal dynamics such as behaviour, development, and types of comorbidities. In conclusion, research on ASD is still challenging. ‘Bench to bedside’ progress will depend on integrative multidisciplinary approaches between basic scientists and clinical investigators to reveal the pathological mechanism of autism.
